# Cleavage of GSDME by caspase-3 determines lobaplatin-induced pyroptosis in colon cancer cells

**DOI:** 10.1038/s41419-019-1441-4

**Published:** 2019-02-25

**Authors:** Junhui Yu, Shan Li, Jie Qi, Zilu Chen, Yunhua Wu, Jing Guo, Kai Wang, Xuejun Sun, Jianbao Zheng

**Affiliations:** 1 Department of General Surgery, First Affiliated Hospital of Xi’an Jiaotong University, 710061 Xi’an, Shaanxi China; 2 Department of Reproductive Medicine, First Affiliated Hospital of Xi’an Jiaotong University, 710061 Xi’an, Shaanxi China; 3 Second Department of Cardiovascular Medicine, Shaanxi Provincial People’s Hospital, 710068 Xi’an, Shaanxi China

## Abstract

Pyroptosis, a form of programmed cell death (PCD), has garnered increasing attention as it relates to innate immunity and diseases. However, the involvement of pyroptosis in the mechanism by which lobaplatin acts against colorectal cancer (CRC) is unclear. Our study revealed that treatment with lobaplatin reduced the viability of HT-29 and HCT116 cells in a dose-dependent manner. Morphologically, HT-29 and HCT116 cells treated with lobaplatin exhibited microscopic features of cell swelling and large bubbles emerging from the plasma membrane, and transmission electron microscopy (TEM) revealed multiple pores in the membrane. GSDME, rather than GSDMD, was cleaved in lobaplatin-induced pyroptosis in HT-29 and HCT116 cells due to caspase-3 activation. Knocking out GSDME switched lobaplatin-induced cell death from pyroptosis to apoptosis but did not affect lobaplatin-mediated inhibition of growth and tumour formation of HT-29 and HCT116 cells in vivo and in vitro. Further investigation indicates that lobaplatin induced reactive oxygen species (ROS) elevation and JNK phosphorylation. NAC, a ROS scavenger, completely reversed the pyroptosis of lobaplatin-treated HT-29 and HCT116 and JNK phosphorylation. Activated JNK recruited Bax to mitochondria, and thereby stimulated cytochrome c release to cytosol, followed by caspase-3/-9 cleavage and pyroptosis induction. Therefore, in colon cancer cells, GSDME mediates lobaplatin-induced pyroptosis downstream of the ROS/JNK/Bax-mitochondrial apoptotic pathway and caspase-3/-9 activation. Our study indicated that GSDME-dependent pyroptosis is an unrecognized mechanism by which lobaplatin eradicates neoplastic cells, which may have important implications for the clinical application of anticancer therapeutics.

## Introduction

Colorectal cancer (CRC) is one of the most common malignancies, whose incidence rate ranks as the fourth leading cause of cancer death^1^. With the ageing of the population, the changes in the lifestyle and the deterioration of the environment, the incidence of CRC in China has increased year after year and has become one of the most serious malignancies^2^. However, most CRC patients are diagnosed at an advanced stage and cannot undergo surgery as a treatment^3^. Thus, chemotherapy is an important part of the comprehensive treatment for advanced CRC^4^. However, the overall response rate of chemotherapy in CRC patients is unsatisfactory and concurrent with a high incidence of adverse effects^5,6^. Therefore, the precise mechanism by which chemotherapy combats CRC requires further elucidation.

Pyroptosis, a form of programmed cell death (PCD), was discovered in recent years and is characterized by cell swelling and large bubbles emerging from the plasma membrane^7^. The pyroptotic cells release interleukin-1β (IL-1β) and interleukin-18 (IL-18), which recruit inflammatory cells and expand the inflammatory response^8^. Therefore, pyroptosis is inflammation-mediated cell death, which is essentially different from apoptosis^9^, a non-inflammatory form of PCD. Pyroptosis was initially believed to be a general innate immune response in vertebrates^7^. Later, the involvement of pyroptosis was observed in multiple pathophysiological processes and diseases, including atherosclerosis^10^, epilepsy^11^, Alzheimer’s disease^12^ and HIV-1 infection^13^. Caspase-1-mediated pyroptosis plays a critical role in the pathogenesis of HIV by causing CD4^+^ T-cell depletion^13^, and pyroptosis-induced activation of the NLRP1 inflammasome is the leading cause of anthrax toxin-mediated lung injury^14^. Furthermore, Tan et al. demonstrated that NLRP1 inflammasome-induced pyroptosis is involved in symptoms relating to Alzheimer’s disease and epilepsy-induced neurodegeneration^11,12^. Exploring the role of pyroptosis in the pathogenesis of human diseases may provide new ideas and effective therapeutic targets for disease prevention and treatment.

Pyroptosis is mainly stimulated by the activation of the canonical inflammatory caspase-1^15^ and non-canonical caspase-11 (caspase-4/-5 in humans)^16,17^. In canonical inflammasomes, the assembled NLRP3, NLRC4, AIM2, and Pyrin proteins activate and cleave pro-caspase-1 to form active caspase-1^18^. The latter can cleave gasdermin D (GSDMD) into the N-terminal and C-terminal fragments. The N-terminus of GSDMD translocates to the membrane and mediate perforation, which leads to extracellular content infiltration, cell swelling and then pyroptosis^19^. In non-canonical inflammasomes, lipopolysaccharide (LPS) can directly bind to caspase-4/-5/-11^20^. On one hand, active caspase-4/-5/-11 can cleave GSDMD, which mediates cell membrane lysis and cell pyroptosis^8^, and stimulate the NLRP3 inflammasome to activate caspase-1, which produces IL-1β and contributes to its release^21^. On the other hand, active caspase-4/5/11 activates pannexin-1 to cause ATP release, which then causes opening of the membrane channel P2X7, leading to the formation of small pores on the cell membrane and subsequent pyroptosis. Activated Pannexin-1 also activates the NLRP3 inflammasome through K^+^ efflux and ultimately leads to IL-1β production and release^22^.

GSDME/DFNA5 (deafness, autosomal dominant 5), a gene associated with autosomal dominant nonsyndromic deafness^23^, was newly identified as a promoter of pyroptosis owing to its cleavage by caspase-3^24^. As a member of the gasdermin superfamily, GSDME shares 28% identity with the region of the pore-forming domain of GSDMD^24^. Genetic mutations within intron 7 of the human GSDME gene led to the skipping of exon 8 and the translation of a C-terminally truncated protein, causing hearing loss^25^. Recently, the role of GSDME in the pathogenesis of human malignancies has attracted increasing attention. GSDME inactivation due to hypermethylation of the promoter was detected in 50% of primary gastric cancers and supports the notion of GSDME as a putative tumour suppressor^26^. Moreover, loss of GSDME has been associated with resistance to etoposide in melanoma cells^27^. Masuda et al. reported that GSDME can be transcriptionally activated by p53 in response to DNA damage caused by etoposide^28^. These studies indicated that the absence of GSDME in tumours might trigger drug resistance.

Lobaplatin (chemical formula: C9H18N2O3Pt), a third-generation platinum anti-neoplastic agent, exerts stronger anti-neoplastic effects with fewer adverse effects. Nowadays, there are a few studies focused on the effect of lobaplatin on human cancer worldwide^29–31^. However, the inflammatory features of lobaplatin in cancer therapy has not ever reported. In the present study, the type of cell death induced in lobaplatin-treated HT-29 and HCT116 cells was investigated. Moreover, the role of GSDME in cell death was further determined. Finally, the mechanism underlying the cleavage of GSDME was explored. Our study aimed to offer new insights into the mechanism by which lobaplatin mediates cell death in CRC.

## Materials and methods

### Cell cultures

HT-29 and HCT116 cells (Shanghai Institute of Cell Biology, Chinese Academy of Sciences) were maintained in RPMI 1640 medium (Gibco BRL, Carlsbad, CA, USA) supplemented with 10% FBS (Gibco BRL, Carlsbad, CA, USA) and incubated at 5% CO_2_ at 37 °C. Once the cells reached 70% confluency, they were incubated in the presence or absence of the caspase-3 specific inhibitor zDEVD-FMK (20 µM, R&D Systems, Minneapolis, MN, USA), the caspase-1 specific inhibitor Z-YVAD-FMK (10 µM, abcam, Cambridge, MA, USA), the RIPK3 inhibitor GSK’872 (2 µM, Aobious, Gloucester, MA, USA), the Reactive oxygen species (ROS) inhibitor NAC (5 mM, abcam, Cambridge, MA, USA) or the JNK inhibitor SP600125 (40 µM, sellerk, Houston, TX, USA), for 3 h and then treated with different doses of lobaplatin for 8 h.

### CRISPR-Cas9 knockout and siRNA knockdown

The px458-GSDME-KO plasmid was constructed as previously described^32^ and stored in our laboratory. HT-29 and HCT116 cells were seeded at a density of 1 × 10^5^ cells/well in six-well plates and transfected with 2 µg of px458-GSDME-KO plasmid by Lipofectamine™ 2000 (Invitrogen, Carlsbad, CA, USA). After 48 h, cells with stable GFP expression were sorted by flow cytometry (BD Biosciences FACS Aria II), and single cells were seeded in a 96-well plate. After 2–3 weeks, each unique clone was tested for GSDME expression by western blotting analysis.

The lentiviral GSDMD-shRNA vectors and their control vectors were constructed and prepared by GeneChem Co., Ltd. (Shanghai, China). All transfections were performed according to the manufacturer’s instructions.

For siRNA knockdown, HT-29 and HCT116 cells were plated in six-well plates (1 × 105 per well). After 24 h, 2 µg of NLRP3 (Genepharma, shanghai, China), caspase-1 (Invitrogen, Carlsbad, CA, USA, 105278), caspase-3 (Invitrogen, 105090), caspase-6 (Invitrogen, 105021), caspase-7 (Invitrogen, 105279), caspase-9 (Invitrogen, 105024) or control siRNA (Invitrogen, 4404020) was transfected with RNAiMAX reagent (Invitrogen) according to the manufacturer’s instructions. After 72 h, transfected HT-29 and HCT116 cells were treated with lobaplatin and subjected to subsequent analyses.

### Cell viability assays

HT-29 and HT116 cells were seeded into 96-well culture plates with 3000 cells/well and treated with different doses of lobaplatin for 8 h. Cell viability was examined using the Cell Counting Kit-8 assay (CCK8, Dojindo, Tokyo, Japan) according to the manufacturer’s protocol. Each experiment was repeated three times.

### Apoptosis evaluation by flow cytometry and tunnel

HT-29 and HT116 cells transfected with the px458-GSDME-KO plasmid were treated with different doses of lobaplatin for 8 h. For flow cytometry detection. Cells were collected by trypsinisation and washed twice with a phosphate-based buffer. Then cells were labelled with annexin V-PE and 7-aminoactinomycin D (7-AAD, BD, Franklin Lakes, NJ, USA) according to the manufacturer’s protocol. Apoptosis was assessed with flow cytometry (BD, Franklin Lakes, NJ, USA). Each experiment was repeated three times. For tunnel analysis, visualized apoptotic cells were labeled with the In Situ Apoptosis Detection kit (Abcam, Cambridge, MA, USA) to detected positive ratio of terminal deoxytransferase-mediated dUTP-biotin nick end labelling (TUNEL) following to the manufacturer’s recommendations.

### RNA isolation and real-time PCR

Total RNA was isolated from cells using TRIzol reagent (Invitrogen, Carlsbad, CA, USA). Complementary DNA (cDNA) was synthesized by the PrimeScript RT Reagent Kit (TaKaRa, Osaka, Japan). Real-time PCR was conducted on an IQ5 instrument (Bio-Rad, CA, USA) using SYBR Green fluorescence signal detection assays (TaKaRa, Osaka, Japan) with primers (Table 1). The specific mRNA expression level was quantified by using the 2^−∆∆CT^ method.

**Table 1:** Primer sequence

Gene	sequence
IL-1β for qRT-PCR	F: 5′- AGCTACGAATCTCCGACCAC -3′
	R: 5′- CGTTATCCCATGTGTCGAAGAA -3′
CASP-1 for qRT-PCR	F: 5′- TTTCCGCAAGGTTCGATTTTCA -3′
	R:5′- GGCATCTGCGCTCTACCATC -3′
NLRP3 for qRT-PCR	F: 5′- CGTGAGTCCCATTAAGATGGAGT -3′
	R: 5′- CCCGACAGTGGATATAGAACAGA -3′
ASC for qRT-PCR	F: 5′- TGGATGCTCTGTACGGGAAG -3′
	R: 5′- CCAGGCTGGTGTGAAACTGAA -3′
GAPDH for qRT-PCR	F: 5′-TGCACCACCAACTGCTTAGC -3′
	R: 5′-GGCATGGACTGTGGTCATGAG -3′

### Protein extraction and western blot

Cells were lysed in RIPA buffer (Heart, Xi’an, China), and 50 μg of total protein from each lysate was then subjected to SDS-PAGE (Beyotime, Shanghai, China), followed by transfer to polyvinylidene fluoride (PVDF) membranes (Millipore, Billerica, MA, USA). The membranes were incubated with primary antibodies targeting NLRP3, ASC, IL-1β, GSDMD, GSDME, caspase-1/-3/-6/-7/-9, Bax, Bcl-2, JNK, p-JNK, Hsp60 and GAPDH (all 1:1000 dilution) at 4 °C overnight. The membrane was then washed four times with TBST buffer for 8 min per wash and incubated with a horseradish peroxidase-conjugated secondary antibody at room temperature for 1 h. Chemiluminescent Horseradish Peroxidase (HRP) substrate (Millipore, Billerica, MA, USA) was added to visualize the protein bands. The antibodies against GAPDH were purchased from Santa Cruz (Dallas, TX, USA), antibodies against NLRP3, ASC, GSDMD, GSDME, and caspase-1/-3/-6/-7/-9 were purchased from Abcam (Cambridge, MA, USA), and antibodies against IL-1β, Bax, Bcl-2 JNK, p-JNK, and Hsp60 were purchased from Cell Signaling Technology (Danvers, MA, USA). Each experiment was repeated three times.

### Measurement of ROS

The ROS levels were measured by a ROS Assay with DCFH-DA (Beyotime, Shanghai, China). After treatment with lobaplatin for 8 h, cells were washed with PBS and stained with DCFH-DA (10 µM) for 30 min at 37 °C in the dark. The level of ROS was determined by flow cytometer (BD, Franklin Lakes, NJ, USA).

### LDH and IL-1β release assay

LDH and IL-1β were measured using a CytoTox96 LDH-release kit (Promega, Madison, WI, USA) and a QuantiCyto IL-1β ELISA kit (Neobioscience, Chenzhen, China) according to the manufacturer’s instructions. The absorbance value at 450 nm was then measured. Each experiment was repeated three times.

### Microscopy imaging

To examine the morphology of apoptotic and pyroptotic cells, cells were first seeded in 35-mm culture dishes. Static bright-field images were captured using a Leica XSP-8CA microscope. The pore-forming activity of lobaplatin-induced pyroptosis was examined by transmission electron microscopy (TEM).

### Nude mouse xenograft assay

The use of all animals in this study was approved by the Institutional Animal Care and Use Committee of the First Affiliated Hospital of Xi’an Jiaotong University. Tumour cells (5 × 10^6^) in the logarithmic growth phase were subcutaneously injected into the right flanks of 4-week-old female BALB/c-nude mice (Shanghai SLAC Laboratory Animal Co. Ltd., Shanghai, China). At 6 days after cell injection, nude mice were intraperitoneally injected with lobaplatin (11 mg/kg). The length (a) and width (b) of the tumour were monitored every 3 days using callipers. The tumour volume (V) was calculated as follows: V = ab^2^/2. At the end of the experiment, the mice were sacrificed, and the xenograft tumours were measured. Apoptosis in the xenograft tumours was detected by flow cytometry (BD, Franklin Lakes, NJ, USA).

### Statistical analysis

Each experiment was repeated three times. Data are presented as the means ± SD. Student’s *t*-test or one-way ANOVA was performed to compare the differences among the groups. Statistical analyses were performed with SPSS 18.0 software (SPSS Inc., Chicago, IL, USA). *P* < 0.05 was considered statistically significant.

## Results

### Lobaplatin inhibits cell viability and induces pyroptosis in colon cancer cells

**Fig. 1:**
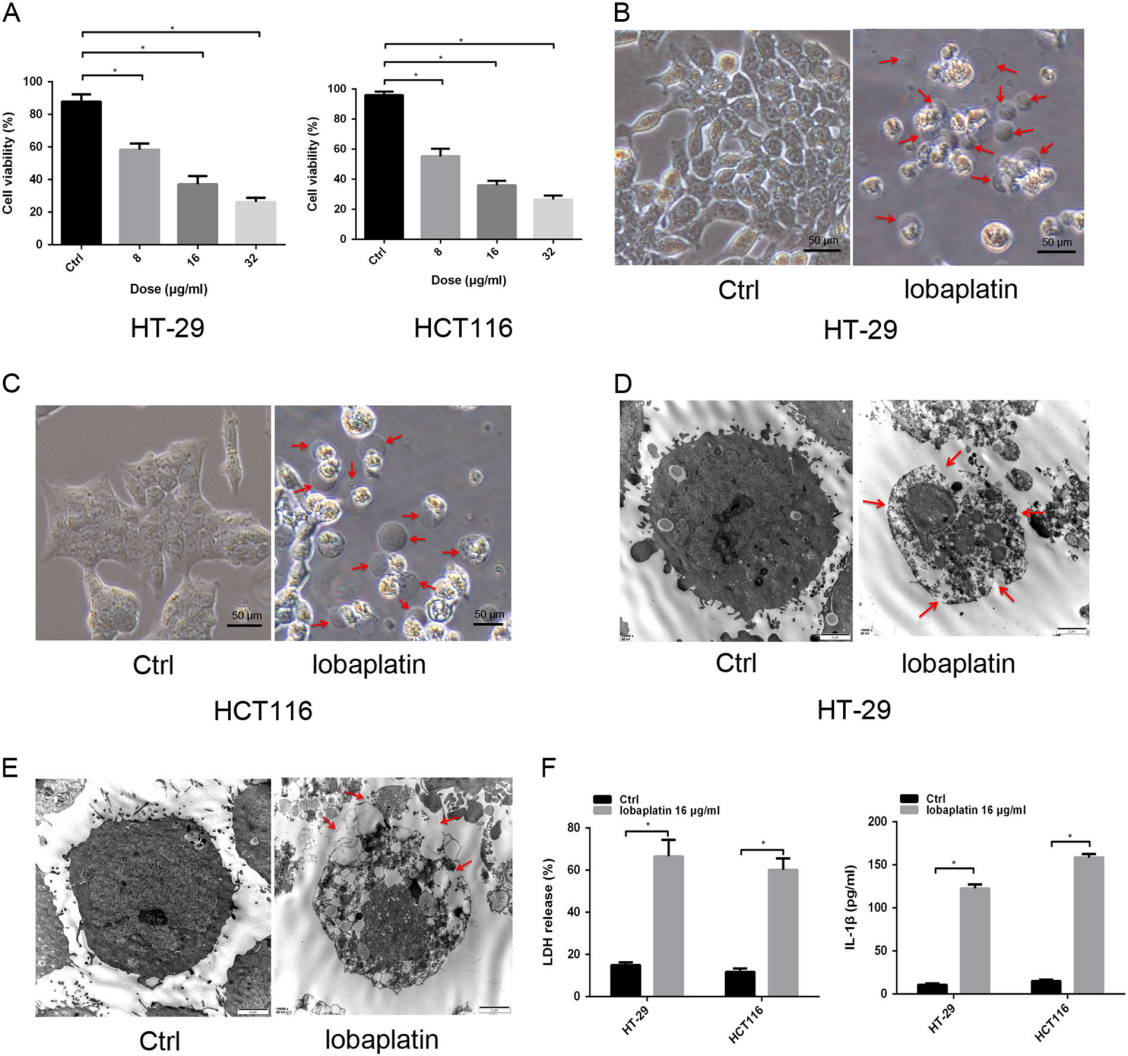
Lobaplatin inhibits cell growth and induces cell pyroptosis in colon cancer cells. HT-29 and HCT116 cells were treated with different doses (0, 8, 16 and 32 μg/ml) of lobaplatin for 8 h. Cell viability was then determined using the CCK8 assay (**a**). Representative bright-field (**b**, **c**) and transmission electron microscopy (**d**, **e**) images of HT-29 and HCT116 cells treated with 16 μg/ml lobaplatin for 8 h. Red arrowheads indicate the large bubbles emerging from the plasma membrane. Scale bar 50 μm. The release of LDH and IL-1β from lobaplatin-treated HT-29 and HCT116 cells was measured by ELISA (**f**). All the data are presented as the mean ± SD from three independent experiments. *p < 0.05 vs. control using one-way ANOVA

To determine the viability of colon cancer cells treated with lobaplatin, HT-29 and HCT116 cells were treated with different doses of lobaplatin for 8 h, and CCK8 assays were performed. Our study showed that treatment with lobaplatin in HT-29 and HCT116 cells inhibited cell viability in a dose-dependent manner (Fig. 1a). Morphologically, lobaplatin-treated HT-29 and HCT116 cells exhibited large bubbles emerging from the plasma membrane and cell swelling (Fig. 1b, c), which highly resembled the pyroptosis induced by the N-terminus of GSDMD^8^. TEM revealed multiple pores formed in the membranes of lobaplatin-treated HT-29 and HCT116 cells (Fig. 1d, e). Cells undergoing necroptosis also showed membrane disruption, cell swelling and lysis^33^. To distinguish pyroptosis from necroptosis, the necroptosis inhibitor GSK’872 was used to block the necroptotic pathway. The results showed that treatment with GSK’872 did not prevent cell death (Supplementary Fig. 1a), indicating that the cell death triggered by lobaplatin in our study is not necroptosis. These data suggest that lobaplatin induces pyroptosis in HT-29 and HCT116 cells.

**Fig. 2:**
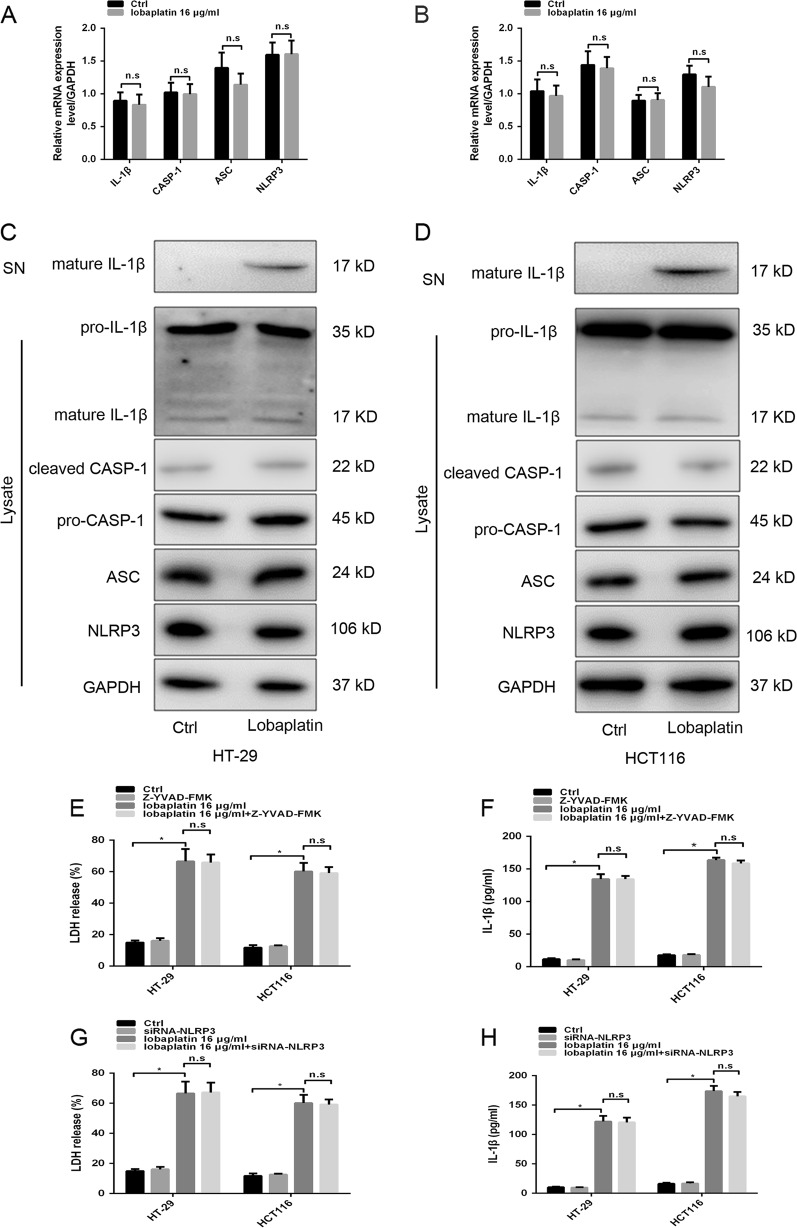
Lobaplatin induced mature IL-1β release independent of both signal 1 and 2 of the NLRP3 inflammasome. The mRNA levels of IL-1β, CASP-1, ASC and NLRP3 in HT-29 (**a**) and HCT116 (**b**) cells treated with lobaplatin (16 μg/ml) for 8 h was detected by qRT-PCR. Supernatants (SN) and cell extracts (Lysate) were analyzed by western blotting analysis (**c**, **d**). ELISA for IL-1β and LDH release of HT-29 (**e**, **f**) and HCT116 (**g**, **h**) cells treated with lobaplatin in the presence or absence of Z-YVAD-FMK or siRNA-NLPR3 transfection. All the data are presented as the mean ± SD from three independent experiments. *p < 0.05 vs. control using one-way ANOVA

Moreover, the release of proinflammatory factors, including IL-1β and LDH, was elevated in lobaplatin-treated HT-29 and HCT116 cells, indicating plasma membrane rupture and leakage (Fig. 1f). The release of IL-1β in pyroptosis requires two signalling of NLRP3 inflammasome: one that upregulates the mRNA of IL-1β and other components (Signal 1) and the other that induces NLPR3 inflammasome activation (Signal 2)^34^. Teatment with lobaplatin did not affect the mRNA level of IL-1β, CASP-1, ASC, and NLRP3 in HT-29 and HCT116 cells (Fig. 2a, b). We next determined whether lobaplatin activates signal 2 of the NLRP3 inflammasome in HT-29 and HCT116 cells. Lobaplatin promoted the release of IL-1β but did not influence the protein of NLRP3, ASC, caspase-1 (cleaved and pro) and pro-IL-1β (mature and pro)(Fig. 2c, d). Furthermore, Z-YVAD-FMK, an inhibitor of caspase-1, or knockdown of NLPR3 (Supplementary Fig. 6a), failed to reverse the release of IL-1β and LDH induced by lobaplatin (Fig. 2e–h). Therefore, lobaplatin induced mature IL-1β release independent of both signal 1 and 2 of the NLRP3 inflammasome, and it is likely that other mechanism may contribute to the effect.

### GSDME but not GSDMD is cleaved in lobaplatin-induced pyroptosis in colon cancer cells by activated caspase-3

**Fig. 3:**
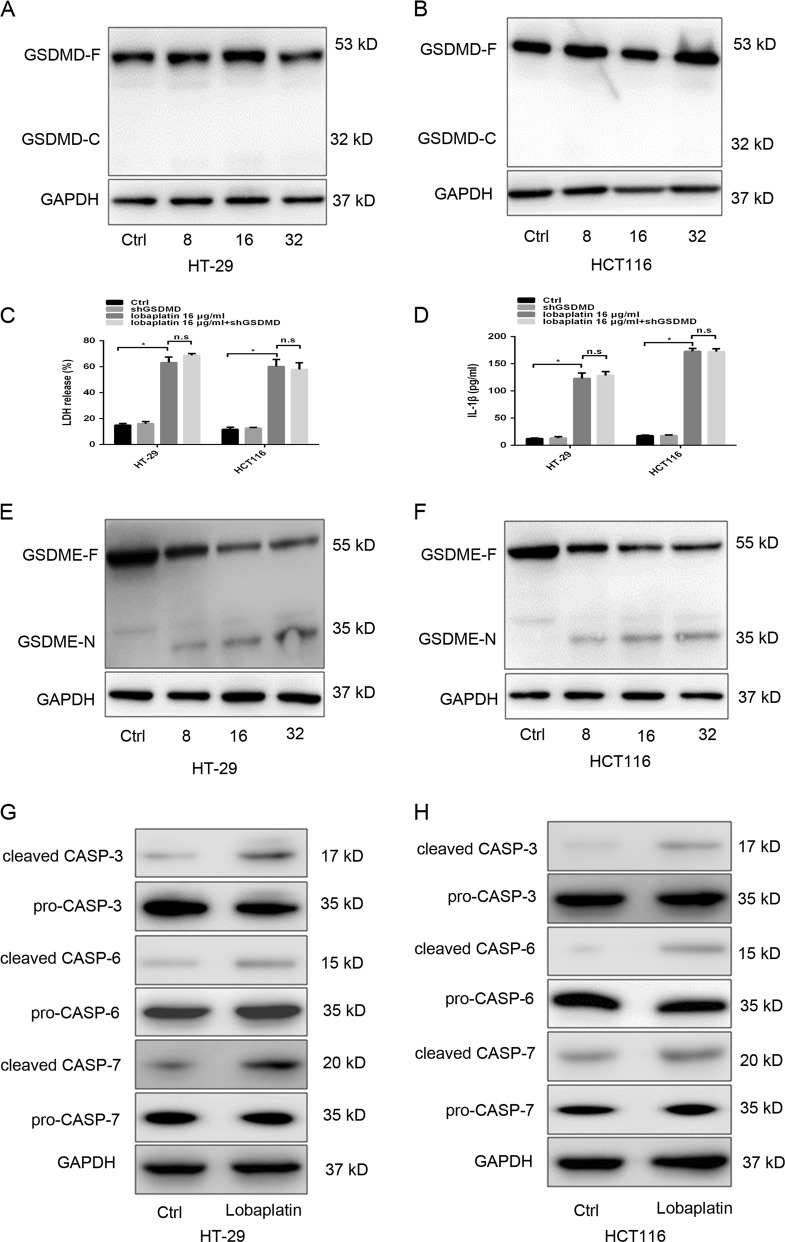
GSDME, rather than GSDMD, is cleaved during lobaplatin-induced pyroptosis in colon cancer cells. Gel images of GSDMD-C terminus (GSDMD-C) in HT-29 and HCT116 cells treated with different doses (0, 8, 16 and 32 μg/ml) of lobaplatin (**a**, **b**). ELISA for IL-1β (**c**) and LDH (**d**) release of HT-29 and HCT116 cells treated with lobaplatin in the presence or absence of GSDMD knockdown. Gel images of GSDME-N in HT-29 (**e**) and HCT116 (**f**) cells treated with different doses (0, 8, 16 and 32 μg/ml) of lobaplatin. Gel images of pro-caspase-3/6/7 and cleaved-caspase-3/6/7 in HT-29 (**g**) and HCT116 (**h**) cells treated with of lobaplatin (16 μg/ml). All the data are presented as the mean ± SD from three independent experiments. *p < 0.05 vs. control using one-way ANOVA

Recently, pyroptosis was shown to be triggered by the N-terminal domain of GSDMD due to cleavage by caspase-1/-4/-5/-11^8^. GSDMD were high-expressed in HT-29, HCT116, and Caco-2 cells (Supplementary Fig. 2a). Our study showed that no cleavage of GSDMD was observed in lobaplatin-treated HT-29 and HCT116 cells (Fig. 3a, b). Furthermore, knockdown of GSDMD (Supplementary Fig. 6b) did not affect the release of IL-1β and LDH (Fig. 3c, d). Therefore, GSDMD is not involved in lobaplatin-induced pyroptosis in HT-29 and HCT116 cells.

Because the pyroptotic gasdermin N-terminus is shared by all gasdermins except for DFNB59^19^, we hypothesized that other GSDMD-related family members can trigger pyroptosis. GSDME has been shown to execute pyroptosis under intrinsic and extrinsic stimulus owing to cleavage by caspase-3^24^. The protein levels of GSDME were high in HT-29 and HCT116 cells but absent in Caco-2 cells (Supplementary Fig. 2b). Our study found that lobaplatin treatment led to the elevated levels of the N-terminal fragment of GSDME in HT-29 and HCT116 cells in a dose-dependent manner (Fig. 3e, f). GSK’872 did not inhibit the generation of the N-terminal fragment of GSDME (Supplementary Fig. 1b and c), further confirming that the cell death triggered by lobaplatin in our study is pyroptosis. Taken together, these results indicated that GSDME is cleaved in lobaplatin-induced pyroptosis in colon cancer cells.

**Fig. 4:**
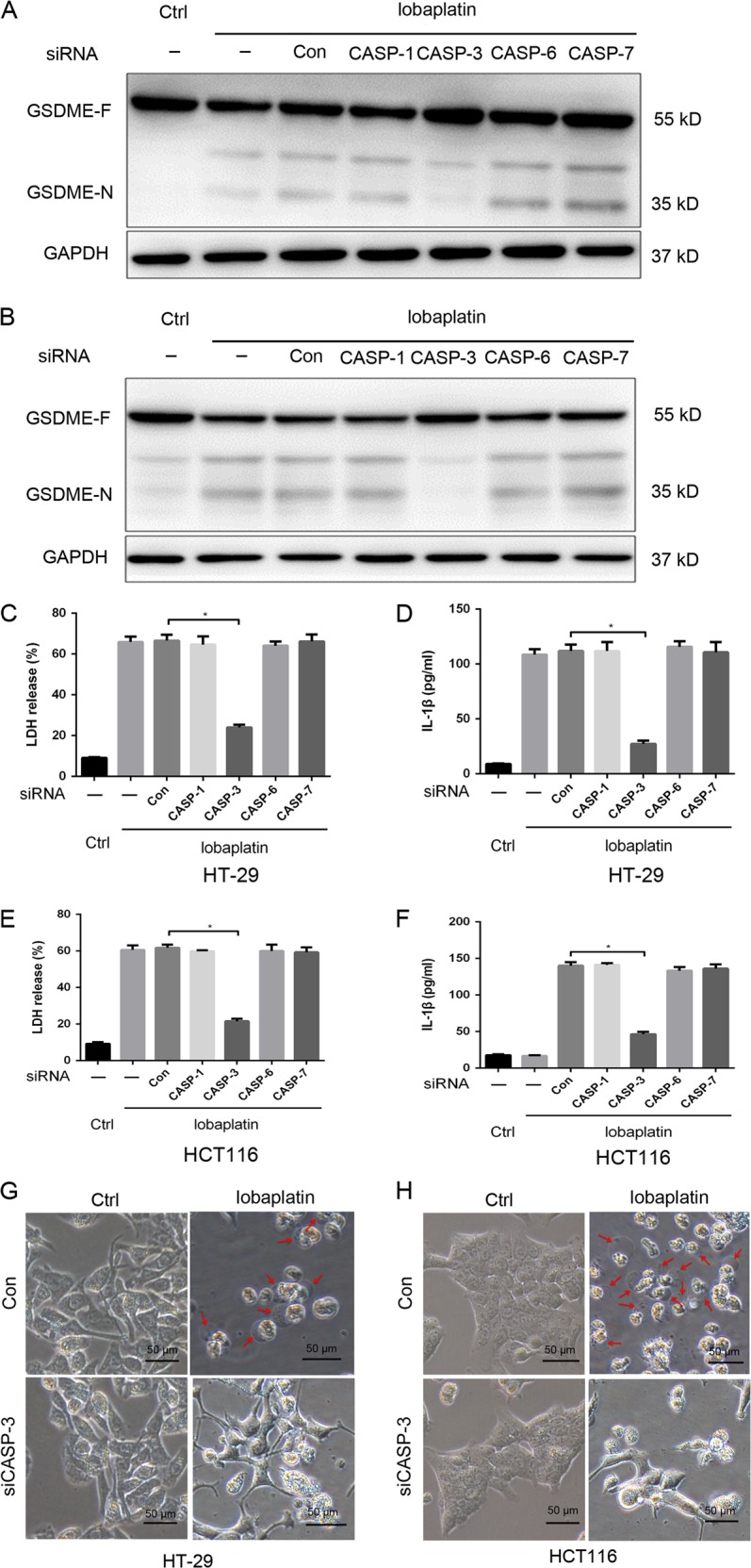
Caspase-3 is required for the cleavage of GSDME. HT-29 and HCT116 cells were transfected with siRNA targeting CASP-1/-3/-6/-7 or control siRNA and then treated with lobaplatin (16 μg/ml) at 8 h after transfection. GSDME-N terminus were detected by western blot. Gel images of GSDME-N in HT-29 (**a**) and HCT116 (**b**) cells. The release of LDH and IL-1β from lobaplatin-treated HT-29 (**c**, **d**) and HCT116 (**e**, **f**) cells was measured by ELISA. Representative bright-field microscopy images of HT-29 (**g**) and HCT116 (**h**) cells treated with lobaplatin in the presence or absence of siRNA-CASP-3 transfecton. Red arrowheads indicate large bubbles emerging from the plasma membrane. Scale bar 50 μm. All the data are presented as the mean ± SD from three independent experiments. *p < 0.05 vs. control using one-way ANOVA

**Fig. 5:**
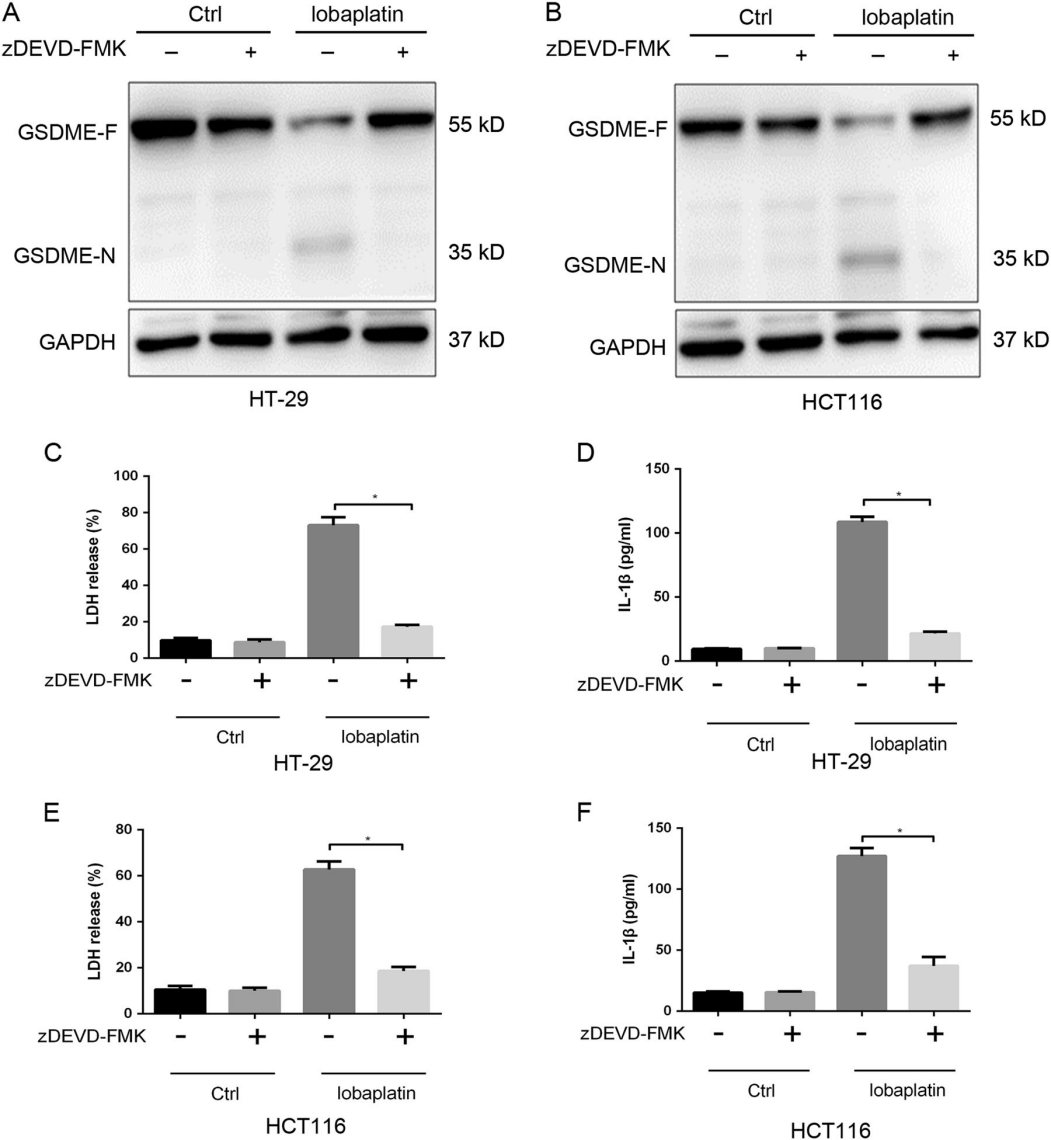
The caspase-3-specific inhibitor zDEVD-FMK inhibits the cleavage of GSDME. Gel images of GSDME-F and GSDME-N expression levels in each group of HT-29 (**a**) and HCT116 (**b**) cells treated with lobaplatin in the presence or absence of zDEVD-FMK (30 µM). The release of LDH and IL-1β and from HT-29 (**c**, **d**) and HCT116 (**e**, **f**) cells was measured by ELISA. All the data are presented as the mean ± SD from three independent experiments. *p < 0.05 vs. control using one-way ANOVA

Furthermore, the levels of active caspase-3/-6/-7 were elevated in response to lobaplatin treatment (Fig. 3g, h), indicating that GSDME may be cleaved by the active caspase. To verify the involvement of caspase in lobaplatin-triggered pyroptosis, we employed siRNA technology to knock down the expression of caspase-1/-3/-6/-7 in HT-29 and HCT116 cells (Supplementary Fig. 6c). We found that caspase-1/-6/-7 knockdown did not affect the lobaplatin-induced cleavage of GSDME (Fig. 4a, b). However, caspase-3 knockdown suppressed the generation of the N-terminal fragment of GSDME. The release of LDH and IL-1β was markedly decreased upon caspase-3 knockdown (Fig. 4c–f) but was not changed upon caspase-1/-6/-7 knockdown. The pyroptotic morphological features were abrogated in HT-29 and HCT116 cells with caspase-3 knockdown (Fig. 4g, h). Furthermore, HT-29 and HCT116 cells were pre-treated with the caspase-3-specific inhibitor zDEVD-FMK and then treated with lobaplatin. We found that zDEVD-FMK treatment inhibited the cleavage of GSDME (Fig. 5a, b) and the release of LDH and IL-1β (Fig. 5c–f) triggered by lobaplatin.

To investigate the critical role of GSDME in pyroptosis, Caco-2 cells with deficient GSDME were treated with lobaplatin. As expected, Caco-2 underwent apoptosis but not pyroptosis in response to lobaplatin (Supplementary Fig. 2c). The nature of cell death was confirmed by the low percentage of annexin V and 7-AAD double-positive cells and the low levels or released LDH and IL-1β (Supplementary Fig. 2d and e).

To test whether GSDME-dependent pyroptosis induction is a general mechanism, HT-29 and HCT116 cells were treated with LPS, which were reported to induce pyroptosis^8^. Our study found that treatment with LPS in HT-29 and HCT116 cells induced the typical morphology of pyroptosis and the release of LDH and IL-1β (Supplementary Fig. 3a–d). However, GSDMD but not GSDME was cleaved in HT-29 and HCT116 treated with LPS (Supplementary Fig. 3e and f). Furthermore, the level of cleaved caspase-1 was elevated in response to LPS. These data indicated that GSDME-mediated pyroptosis induced by lobaplatin is specific.

In summary, these data indicated that GSDME rather than GSDMD is cleaved in lobaplatin-induced pyroptosis in colon cancer cells and that the cleavage of GSDME is due to caspase-3 activation.

### Knocking out GSDME switches lobaplatin-induced cell death from pyroptosis to apoptosis in vitro

**Fig. 6:**
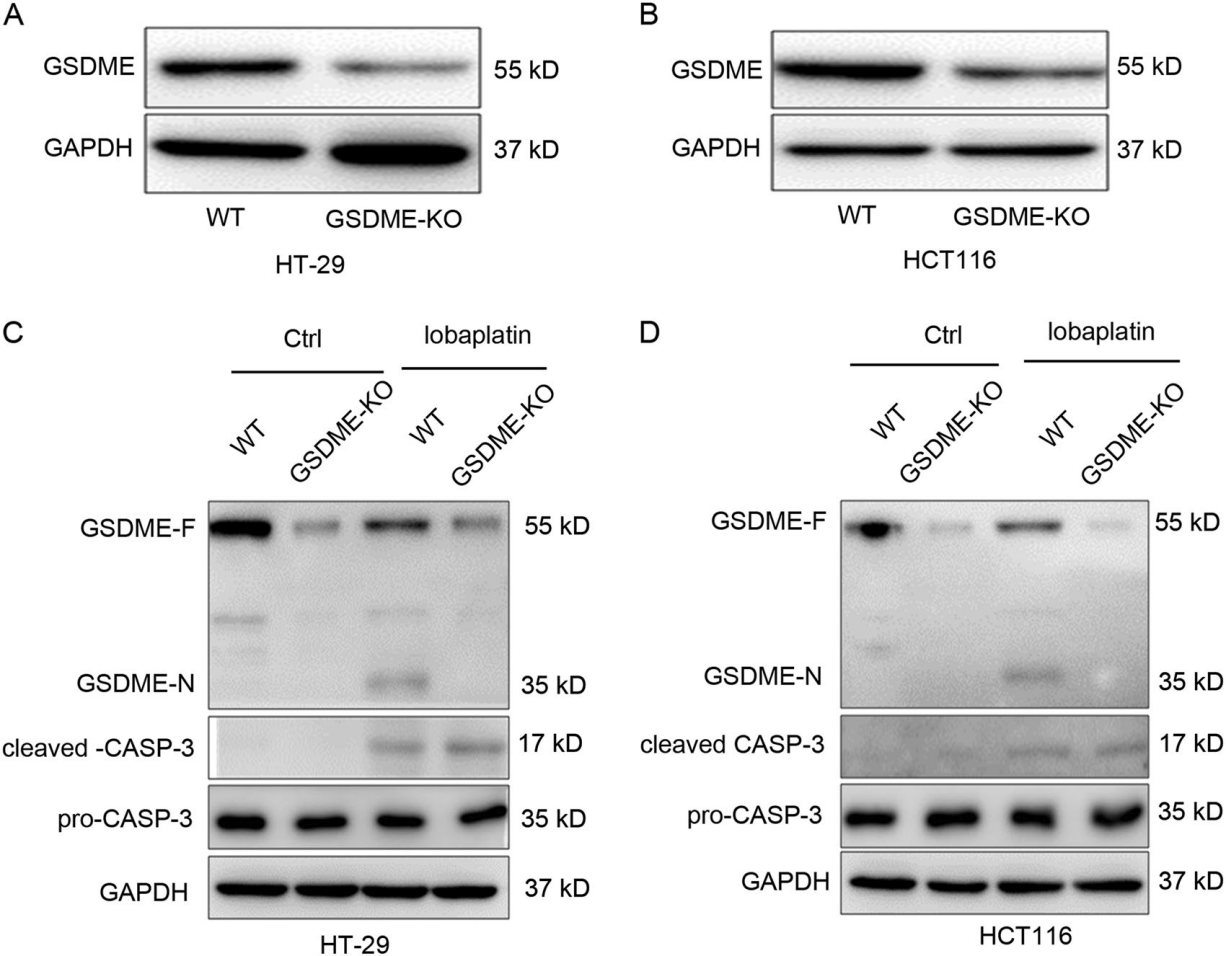
GSDME knockout completely abolished the lobaplatin-mediated release of the N-terminal fragment of GSDME. Gel images of GSDME expression levels in HT-29 (**a**) and HCT116 (**b**) cells transfected with the px458-GSDME-KO plasmid. Gel images of GSDME expression levels in HT-29 (**c**) and HCT116 (**d**) cells treated with lobaplatin in the presence or absence of GSDME knockout. All the data are presented as the mean ± SD from three independent experiments. *p < 0.05 vs. control using one-way ANOVA

**Fig. 7:**
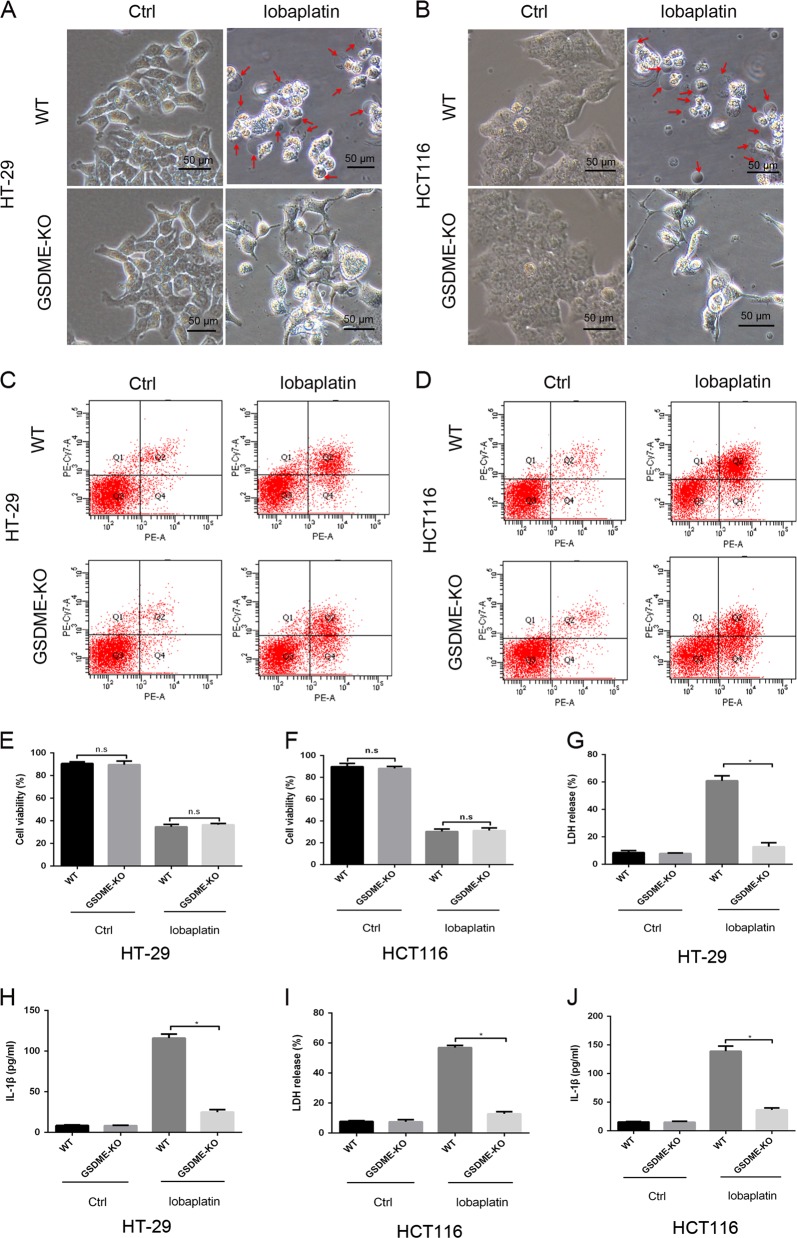
GSDME knockout switches lobaplatin-induced cell death from pyroptosis to apoptosis in vitro. Representative bright-field microscopic images of HT-29 (**a**) and HCT116 (**b**) cells treated with lobaplatin in the presence or absence of GSDME knockout. Red arrowheads indicate large bubbles emerging from the plasma membrane. Scale bar 50 μm. The percentage of annexin V and 7-AAD double-positive cells in HT-29 (**c**) and HCT116 (**d**) was detected by flow cytometry in the WT and GSDME-KO groups. The effect of GSDME knockout on the cell viability of HT-29 (**e**) and HCT116 (**f**) cells in the presence of lobaplatin treatment for 8 h was assessed by the CCK8 assay. The release of LDH and IL-1β from HT-29 (**g**, **h**) and HCT116 (**i**, **j**) cells was measured by ELISA. All the data are presented as the mean ± SD from three independent experiments. *p < 0.05 vs. control using one-way ANOVA

The abovementioned results demonstrated that GSDME is cleaved in lobaplatin-induced pyroptosis in colon cancer cells. Thus, we aimed to investigate whether GSDME is essential for lobaplatin-induced pyroptosis in colon cancer cells. The expression of GSDME in HT-29 and HCT116 cells was knocked out using CRISPR-Cas9 technology (Fig. 6a, b). Western blotting analysis showed that the N-terminal fragment of GSDME was barely detected in GSDME-knockout cells treated with lobaplatin (Fig. 6c, d). However, GSDME knockout did not affect the cleavage of caspase-3 compared with the levels observed in wild-type (WT) cells, which further confirmed that GSDME was downstream of activated caspase-3 in lobaplatin-mediated pyroptosis. Intriguingly, unlike the pyroptotic morphology of lobaplatin-treated WT cells, GSDME-knockout cells exhibited shrinking, fragmentation into apoptotic bodies and non-lysis cell death (Fig. 7a, b), indicating that GSDME knockout switches lobaplatin-induced cell death pyroptosis to apoptosis. Furthermore, the results of the flow cytometry analyses confirmed that knockout GSDME led to fewer annexin V and 7-AAD double-positive cells but more annexin V single-positive cells in HT-29 and HCT116 cells treated with lobaplatin (Fig. 7c, d). However, the tunnel analysis showed that knocking out GSDME did not affect the apoptotic character of cell death induced by lobaplatin (Supplementary Fig. 4a and b), for pyroptosis is associated with DNA fragmentation, which is similar to apoptosis^35^. Intriguingly, knocking out GSDME did not affect the growth of HT-29 and HCT116 cells in the presence of lobaplatin (Fig. 7e, f). In addition, the release of LDH and IL-1β was decreased in GSDME-knockout cells (Fig. 7g–j). Taken together, these data suggest that GSDME is essential for lobaplatin-induced pyroptosis in colon cancer cells and that knocking out GSDME switches lobaplatin-induced cell death from pyroptosis to apoptosis.

### GSDME is activated by the lobaplatin-elevated ROS/JNK signaling

**Fig. 8:**
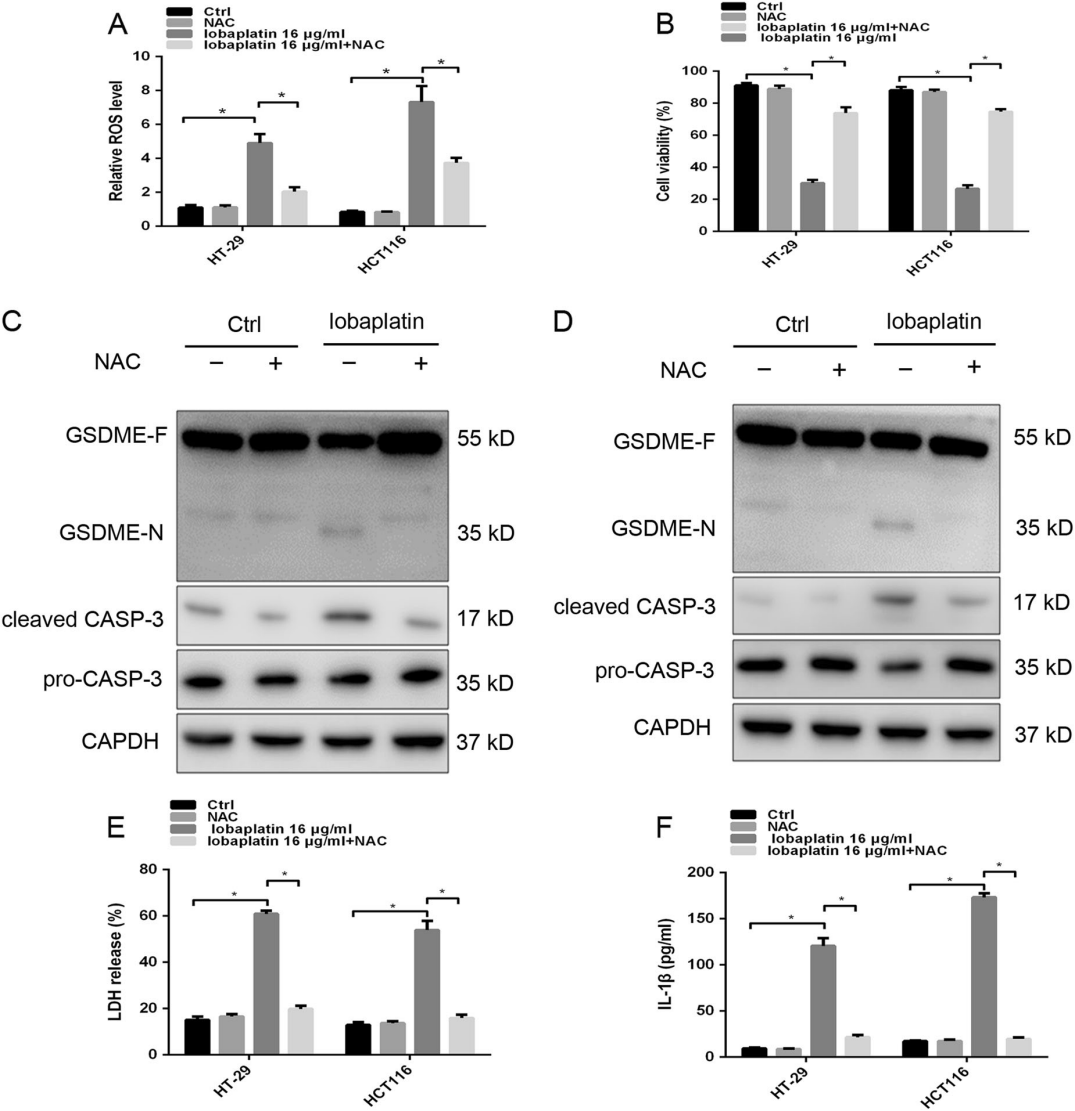
ROS is involved in GSDME-dependent pyroptosis in response to lobaplatin. The ROS levels of HT-29 and HCT116 cells treated with lobaplatin in the presence or absence of NAC were measured by a ROS Assay (**a**). The effect of NAC on the cell viability of HT-29 and HCT116 cells in the presence of lobaplatin treatment for 8 h was assessed by the CCK8 assay (**b**). Gel images of GSDME-N and cleaved CASP-3 in HT-29 (**c**) and HCT116 (**d**) cells treated with lobaplatin (16 μg/ml). The release of LDH (**e**) and IL-1β (**f**) from HT-29 and HCT116 cells was measured by ELISA. All the data are presented as the mean ± SD from three independent experiments. *p < 0.05 vs. control using one-way ANOVA

We sought to explore the underlying molecular mechanism by which GSDME-dependent pyroptosis was triggered upon lobaplatin treatment. Caspase-3 activation can occur via the death receptor pathway or mitochondrial pathway^36,37^. Lobaplatin has been reported to induce gastric cancer cell apoptosis by activating the ROS-dependent mitochondrial pathway^38^. ROS, active forms of oxygen, generates as by-products from cellular metabolism^39^. ROS have been reported to regulate cells apoptosis and autophagy^40^. However, whether lobaplatin-elevated ROS is linked to pyroptosis has not been reported. Our study found that treatment with lobaplatin boosts ROS level of HT-29 and HCT116 cells (Fig. 8a). NAC, an inhibitor of ROS, markedly inhibits the ROS elevation induced by lobaplatin and thereby attenuated cell death (Fig. 8b). Moreover, NAC abolished the cleaved GSDME, active caspase-3 and the release of LDH and IL-1β (Fig. 8c–f). Therefore, ROS is involved in GSDME-dependent pyroptosis in response to lobaplatin.

**Fig. 9:**
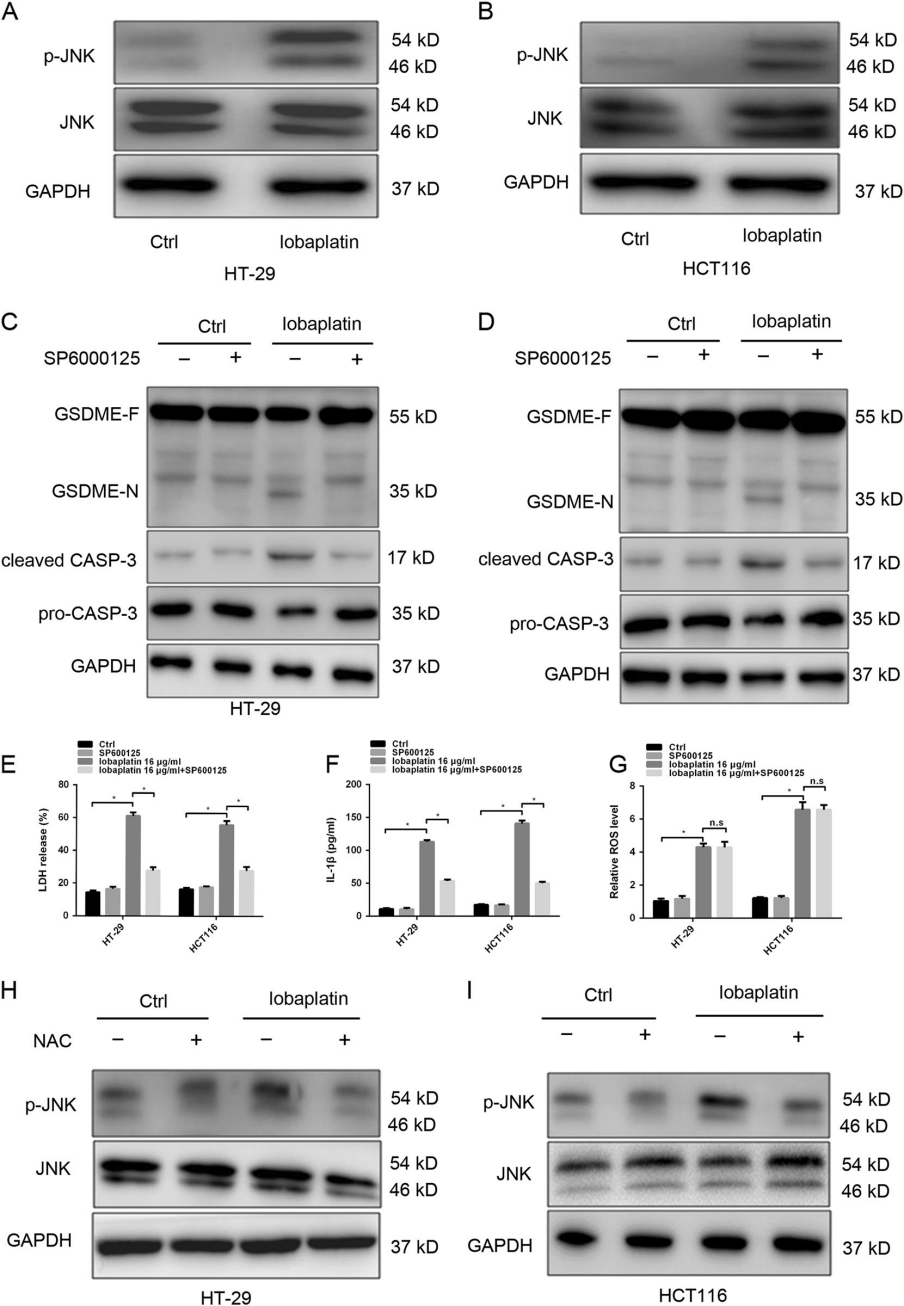
GSDME-dependent pyroptosis is downstream of the ROS/JNK signalling. Gel images of JNK and p-JNK in HT-29 (**a**) and HCT116 (**b**) cells treated with lobaplatin (16 μg/ml). Gel images of GSDME-N and cleaved CASP-3 in HT-29 (**c**) and HCT116 (**d**) cells treated with lobaplatin (16 μg/ml) in the presence or absence of SP6000125. The release of LDH (**e**) and IL-1β (**f**) from HT-29 and HCT116 cells was measured by ELISA. The ROS levels of HT-29 and HCT116 cells treated with lobaplatin in the presence or absence of SP6000125 were measured by a ROS Assay (**g**). Gel images of JNK and p-JNK in HT-29 (**h**) and HCT116 (**i**) cells treated with lobaplatin (16 μg/ml) in the presence or absence of NAC. All the data are presented as the mean ± SD from three independent experiments. *p < 0.05 vs. control using one-way ANOVA

ROS could affect various signaling pathways such as MAPK signalling pathway^41^. More importantly, JNK, a stress-activated protein kinase (SAPK) of the MAPK family, plays a pivotal role in many cellular events, including apoptosis and autophagy^42^. Our study found that lobaplatin stimulation markedly elevated the phosphorylation of JNK in HT-29 and HCT116 cell (Fig. 9a, b). More importantly, JNK inhibitor, SP600125, effectively abolished the cleaved GSDME, active caspase-3 and the release of LDH and IL-1β (Fig. 9c–f). Therefore, JNK is likely an upstream regulator of GSDME-dependent pyroptosis. Furthermore, JNK inhibitor treatment did not influence the lobaplatin-induced ROS elevation (Fig. 9g). In contrast, lobaplatin-induced phosphorylation of JNK was abolished by the ROS scavengers NAC (Fig. 9h, i). Altogether, JNK, which is a novel downstream factor of ROS, likely senses lobaplatin-elevated ROS signalling, induce pyroptosis via cleavage of GSDME by caspase-3 activation.

### GSDME-dependent pyroptosis is downstream of the ROS/JNK/Bax-mitochondrial apoptotic pathway

**Fig. 10:**
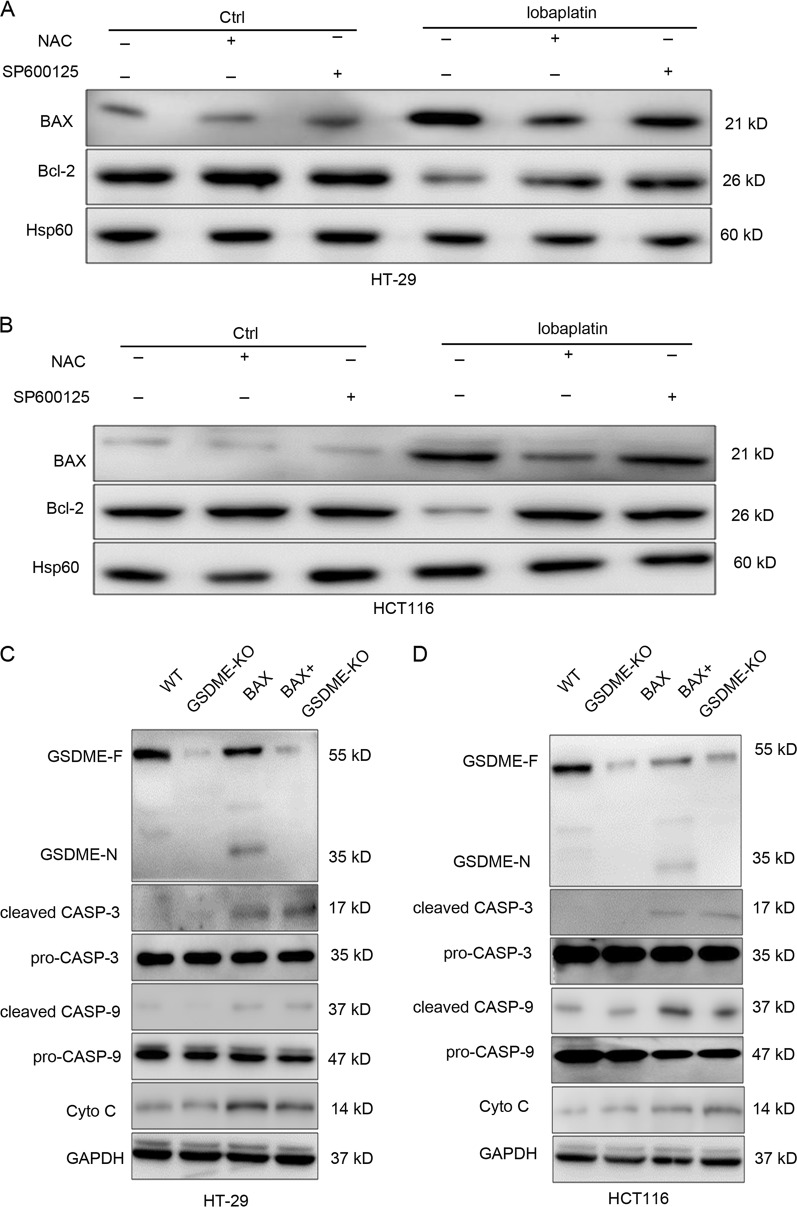
Overexpression of Bax led to an increase in the levels of cleaved GSDME and cleaved caspase-3 in HT-29 and HCT116 cells. Gel images of Bcl-2 and BAX expression levels in HT-29 (**a**) and HCT116 (**b**) cells treated with lobaplatin in the presence or absence of NAC or SP6000125. Gel images of GSDME-N and cleaved CASP-3/-9 expression levels in HT-29 (**c**) and HCT116 (**d**) cells transfected with BAX overexpression plasmid in the presence or absence of GSDME knockout. All the data are presented as the mean ± SD from three independent experiments. *p < 0.05 vs. control using one-way ANOVA

**Fig. 11:**
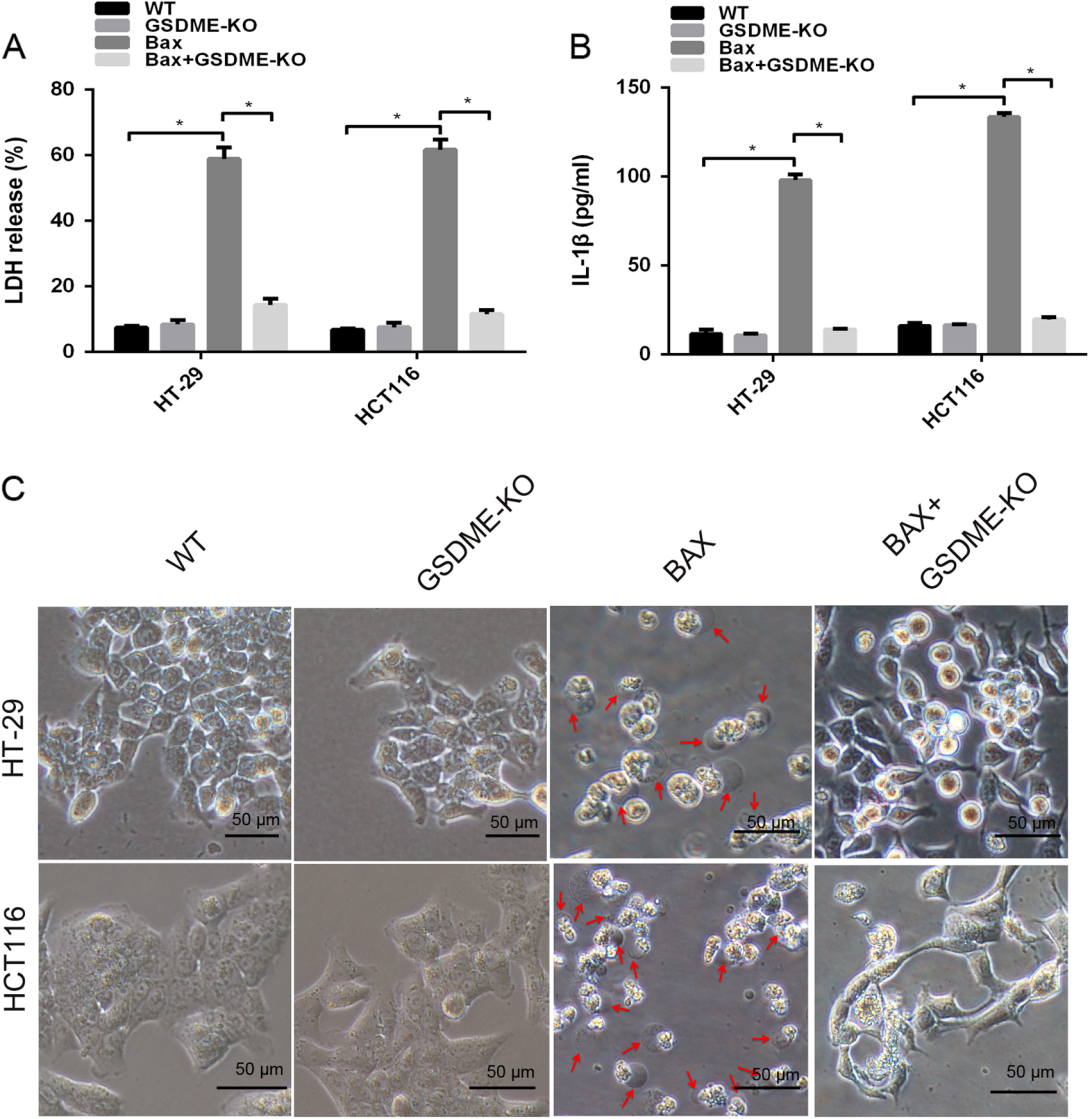
Bax overexpression showed morphology characteristic of pyroptosis. The release of LDH (**a**) and IL-1β (**b**) from HT-29 and HCT116 cells transfected with the BAX overexpression plasmid in the presence or absence of GSDME knockout was measured by ELISA. Representative bright-field microscopy images of HT-29 and HCT116 cells transfected with the BAX overexpression plasmid in the presence or absence of GSDME knockout (**c**). Red arrowheads indicate large bubbles emerging from the plasma membrane. Scale bar, 50 μm. All the data are presented as the mean ± SD from three independent experiments. *p < 0.05 vs. control using one-way ANOVA

The ROS/JNK signalling has been reported to be important for Bax-mitochondrial apoptotic pathway^43,44^. Thus, we investigate whether Bax is involved in lobaplatin-induced pyroptosis via ROS/JNK signalling. Lobaplatin treatment downregulated the protein levels of Bax and upregulated the protein levels of Bcl-2 (Fig. 10a, b), which can be significantly reversed by ROS inhibitor NAC or JNK inhibitor SP600125. To further confirm the role of Bax in lobaplatin-induced cell death, we constructed Bax-EGFR constructs and transfected them into HT-29 and HCT116 cells (Supplementary Fig. 6d). Overexpression of Bax resulted in characteristic pyroptotic phenotype (Fig. 11c) and elevated LDH and IL-1β release (Fig. 11a, b). Western blotting analysis showed that the N-terminal fragment of GSDME, cleaved caspase-3/-9 and the release of cytochrome c were induced in response to Bax overexpression (Fig. 10c, d). Furthermore, GSDME knockout eliminated the pyroptotic morphology and LDH and IL-1β release (Fig. 11a–c) but did not affected the expression of cleaved caspase-3/-9 and cytochrome c (Fig. 10c, d). Caspase-9 is reported to cleave and activate caspase-3^45^. As expected, knockdown of caspase-9 (Supplementary Fig. 6e) led to the blockade of GSDME and caspase-3 cleavage, and LDH and IL-1β release induced by Bax overexpression (Supplementary Fig. 5a–d). Taken together, these results showed that GSDME mediates pyroptosis downstream of the ROS/JNK/Bax-mitochondrial apoptotic pathway and caspase-3/-9 activation.

In Caco-2 cells with deficient GSDME expression, overexpression of Bax increased the levels of cleaved caspase-3/-9 (Supplementary Fig. 2f) but failed to induce pyroptosis and LDH and IL-1β release (Supplementary Fig. 2g and h). These data further revealed that GSDME is necessary for Bax-mitochondrial apoptotic pathway- induced pyroptosis.

### GSDME knockout did not affect lobaplatin-inhibited tumour formation of colon cancer cells in vivo

**Fig. 12:**
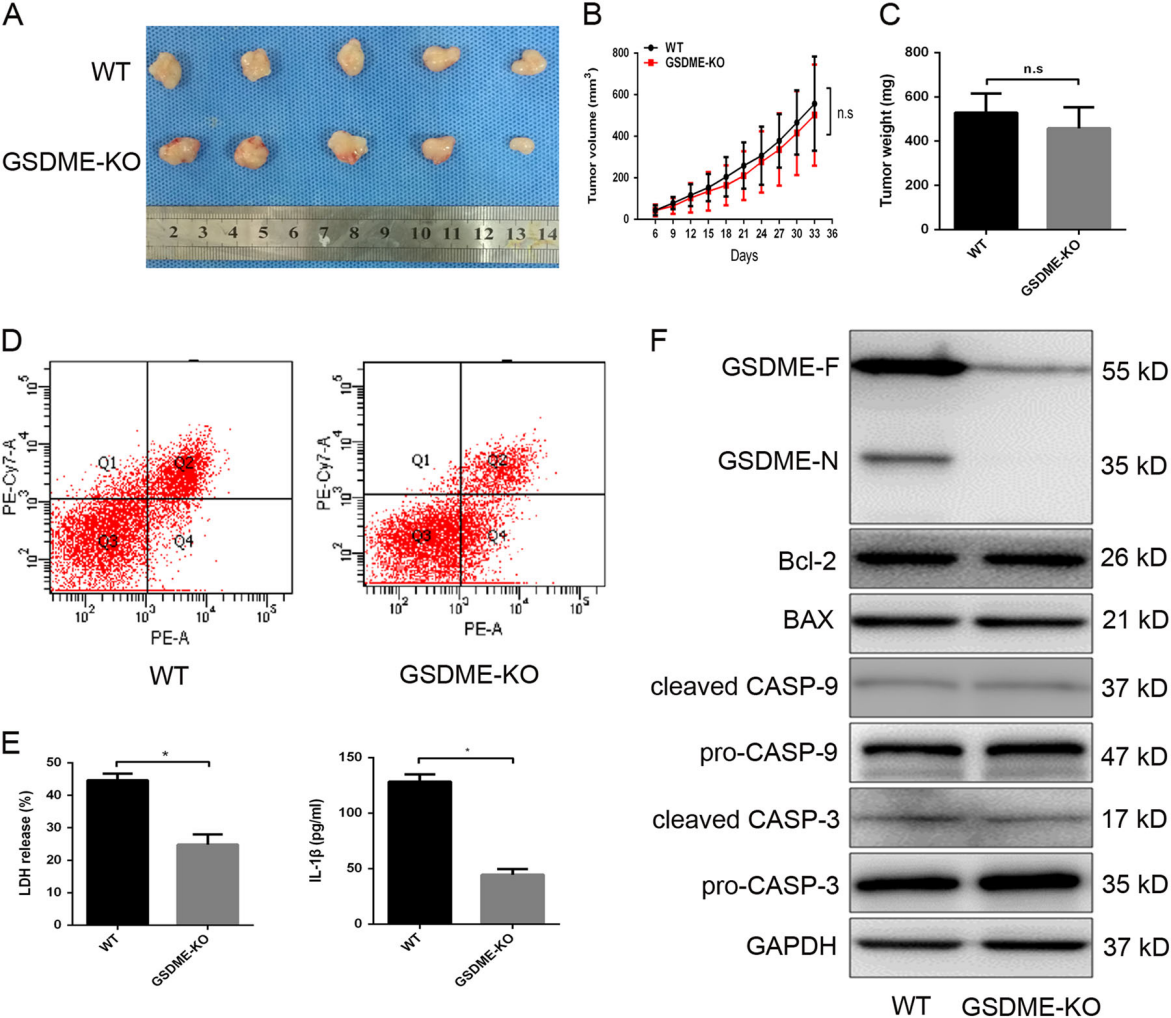
GSDME knockout did not affect lobaplatin-mediated inhibition of tumour formation in vivo. Image of xenograft tumours formed by HT-29 cells treated with lobaplatin in the presence or absence of GSDME knockout (**a**). Tumour growth curves (**b**) and tumour weights (**c**) are shown for the HT-29-GSDME-KO and HT-29-WT groups. The percentage of annexin V and 7-AAD double-positive HT-29 cells in the GSDME-KO and WT groups was detected by flow cytometry (**d**). The release of serum LDH and IL-1β in the GSDME-KO and WT nude mice was measured by ELISA (**e**). Gel images of GSDME-N, Bcl-2, BAX and cleaved CASP-3/-9 expression levels in the HT-29-GSDME-KO and HT-29-WT groups (**f**). All the data are presented as the mean ± SD from three independent experiments. *p < 0.05 vs. control using Student’s *t*-test

To further explore the role of GSDME-mediated pyroptosis in tumour formation upon lobaplatin treatment, GSDME-modified HT-29 cells and their control cells were subcutaneously injected into nude mice. After 4 weeks post-injection, we treated nude mice with lobaplatin via intraperitoneal injection. The results showed that the size and weight of the xenograft tumours in the GSDME-knockout group were comparable to those in the WT group (Fig. 12a–c). Furthermore, flow cytometry analyses showed that GSDME-knockout tumour tissues had a higher percentage of annexin V single-positive cells and fewer annexin V and 7-AAD double-positive cells (Fig. 12d). Consistent with the in vitro result of tunnel analysis, GSDME knockout did not affect the apoptotic character of cell death in tumour tissues treated with lobaplatin (Supplementary Fig. 4c). The release of serum LDH and IL-1β in nude mice was decreased in the GSDME knockout group compared with that in the WT group (Fig. 12e), which indicated that GSDME knockout attenuates the inflammation induced by lobaplatin. Moreover, the release of the N-terminal fragment of GSDME was barely detected in the knockout group (Fig. 12f). However, GSDME knockout did not affect the expression of Bax, Bcl-2 or cleaved caspase-3/-9, indicating that the activity of GSDME in mediating pyroptosis is downstream of the mitochondrial apoptotic pathway and caspase-3/-9 activation.

Taken together, these results suggest that GSDME knockout switches lobaplatin-induced pyroptosis to apoptosis but does not affect tumour formation in colon cancer cells treated with lobaplatin.

## Discussion

Pyroptosis was previously referred to as caspase-1-induced monocyte death in response to certain viral or bacterial infections^13^. Later, the discovery that caspase-4, -5 and -11 induces pyroptosis in non-monocytic cells upon treatment with LPS expanded the concept of pyroptosis and indicates that pyroptosis is not cell type specific^46^. Recently, Alnemri and his colleagues demonstrated that treatment with certain apoptotic stimuli activated caspase-3 cleaves GSDME and triggers secondary necrosis after apoptosis or pyroptosis^24^, which challenges a long-standing view that chemotherapy act most potently through stimulating apoptosis. So far, whether pyroptosis is involved in chemotherapy of lobaplatin in CRC remains unclear. In the present study, we demonstrated that lobaplatin induces ROS/JNK signalling to induce the pyroptosis of colon cancer cells via a novel Bax-caspase-GSDME pathway. In colon cancer cells, lobaplatin-elevated ROS caused the phosphorylation of JNK. Activated JNK further enhanced Bax translocation to the mitochondria, consequently activating caspase-3, and ultimately inducing the cleavage of GSDME and pyroptosis of colon cancer cells. Our study shed light on the interrelation between chemotherapy drug and pyroptosis, and proposed a functional role for pyroptosis in antitumour treatment.

Lobaplatin was reported to induce cell death by elevating ROS^30^. Excessive generation of ROS could interfere with cellular signaling pathways, and JNK also has a pivotal role in many cellular events^47^. However, the underlying molecular mechanism of the lobaplatin-induced pyroptosis remains unclear. Our study found lobaplatin induced ROS elevation and JNK phosphorylation. NAC, a ROS scavenger, completely reversed the pyroptosis of lobaplatin-treated HT-29 and HCT116 and the JNK phosphorylation. However, ROS generation was not attenuated by the JNK inhibitor, implying that ROS is the proximal event of JNK. Activated JNK recruited Bax to mitochondria, and thereby stimulated cytochrome c release to cytosol, followed by caspase-3 cleavage and pyroptosis induction. ROS often regulate protein functions via oxidation of cysteines^48^. Zhou et al. found that the outer mitochondrial membrane protein Tom 20 can be oxidated by the ROS elevation, subsequently facilitates Bax recruitment to mitochondria and stimulates caspase-3/GSDME-mediated pyroptosis in response to iron treatment^49^. Therefore, besides apoptosis and autophagy^40^, ROS can induce cell death towards pyroptosis pathway. Further study will focus on whether there exists other molecular mechanism of ROS-mediated pyroptosis. From these data, we concluded that lobaplatin induced pyroptosis by Bax-caspase-GSDME pathway via the ROS/JNK signalling.

Necroptosis, a form of programmed necrotic cell death, is activated by a pro-apoptotic stimulus through the receptor-interacting serine/threonine-protein kinase 1 (RIPK1)–RIPK3-mixed lineage kinase domain-like (MLKL) axis^33^. Cells undergoing necroptosis also showed membrane disruption, cell swelling and lysis. The cell death triggered by lobaplatin in our study is pyroptosis but not necroptosis, as demonstrated by the following evidence: (1) necroptosis could not be blocked by caspase inhibitors, but the caspase-3-specific inhibitor zDEVD-FMK markedly inhibited lobaplatin-induced pyroptosis, and caspase activation was responsible for pyroptosis; (2) the RIPK3 inhibitors GSK’872 could not influence the lobaplatin-induced pyroptosis of colon cancer cells; (3) GSDME, an pyroptosis executor, was cleaved due to caspase-3 activation in response to iron stimulation, which was linked to pyroptosis induction. Notably, lobaplatin can stimulate different forms of cell due to the activation of different signaling pathways. In addition to pyroptosis, lobaplatin-induced apoptosis and necroptosis have also been reported^50^.

For a long time, secondary necrosis has been considered as a final phase of PCD following apoptosis completion, indicated by lysis, osmotic cell swelling and the leakage of the cell contents, features that are also common to pyroptosis^51^. Moreover, secondary necrosis resembles pyroptosis in their ability to deliver potent proinflammatory signals. There exists great divergence between secondary necrosis after apoptosis and pyroptosis. On one hand, Alnemri et al. provide convincing evidence in support of the notion that GSDME-mediated pyroptosis was considered the secondary necrosis, for cleavage of GSDME is downstream of mitochondrial apoptotic pathway and caspase-3 activation^24^. On the other hand, GSDME-mediated pyroptosis may precede or even impede apoptosis in cells with high GSDME expression, while GSDME-deficient cells favour apoptotic outcomes^52^. Distinct from these previous conceptions, Lu et al. demonstrated that apoptosis and pyroptosis were contemporaneously instigated by anticancer therapy, considering the biochemical markers for apoptosis and pyroptosis were synchronously detected in drug-treated adherent cells and supernatant cells^53^. However, drug-treated GSDME knockouts yielded more cleaved PARP and caspase-3 products, which was the hallmark of apoptosis. Our study indicated that knocking out GSDME switches lobaplatin-induced cell death from pyroptosis to apoptosis, supporting that the expression level of GSDME determines the form of cell death in caspase-3-activated cells. Considering the heterogeneity of GSDME expression in tumour tissues^53^, we argued it was plausible to speculate that apoptosis and pyroptosis were contemporaneously stimulated by chemotherapy. Besides caspase-3–dependent pyroptosis, recent study indicated the apoptotic caspase-8 induces cleavage of GSDME and GSDMD to elicit pyroptosis during Yersinia infection, which implied that pyroptosis and apoptosis share many signal transduction pathways^54,55^. Further study will be needed to focus on the potential mechanism underlying the transformation between pyroptosis and apoptosis.

Previous study indicated that GSDME expression is generally undetectable in CRC^56^, gastric cancer^26^, breast cancer^57,58^ and hepatocellular carcinoma^59^ due to epigenetic inactivation. Therefore, whether GSDME-dependent pyroptosis as a universal mechanism of chemotherapy to eradicate neoplastic cells remain controversial. Notably, owing to adoption of in vitro systems or invalidated antibodies, previous studies did not always reflect the actual GSDME level of in vivo tumour. In contrast to previous conclusion that no or little GSDME is expressed in other tumour types, Zhuang et al. found that GSDME was ubiquitously expressed among diverse molecular subtypes of lung cancer^53^. Therefore, scientifically-sound studies are still needed to explore the expression partner of GSDME in various type of tumours. Interestingly, GSDME-dependent pyroptosis may be involved in chemotherapy drug-induced toxicity. GSDME^−/−^ mice exhibited reduced adverse effects of chemotherapy drugs, including tissue damage (intestine and lung) and weight loss^24^. The clinical significance of GSDME-dependent pyroptosis in cancer chemotherapy of CRC still requires further investigation.

Whether GSDME-dependent pyroptosis contributes to anti-cancer effects of chemotherapy is determined. In lung cancer, genetic deletion of GSDME promoted drug resistance, while GSDME overexpression led to enhanced drug sensitivity in vivo and in vitro^53^. Lage et al. reported that GSDME expression was decreased in etoposide-resistant melanoma cells and was negatively associated with cell resistance to etoposide-induced cell death^27^. Genetic GSDME deletion attenuated drug response and induce more drug-tolerant state. Reversal of GSDME silencing by treatment with the DNA methyltransferase inhibitor decitabine may sensitize tumour cells to chemotherapy drugs^24^. However, our study indicated that knocking out GSDME switches lobaplatin-induced cell death from pyroptosis to apoptosis but does not affect the growth and tumour formation of colon cancer cells treated with lobaplatin. Consistent with these results, knocking out GSDME has no effect on the growth of gastric cancer cells^32^. There are at least three factor contributing to the notion that the GSDME did not influence on anti-cancer effects of chemotherapy: (1) GSDME levels determines the form of cell death. Either apoptosis or pyroptosis alone was sufficient for driving cell death and modulating GSDME just altered the apoptotic process; (2) in addition to GSDME, other mediator is involved in pyroptosis induction; (3) In the absence of GSDME, other form of cell death may result in eradicating tumour cells. Chemotherapy may trigger different form of programmed cell death (apoptosis, necroptosis or pyroptosis) to synergistically, rather than mutually exclusively, eradicate tumour cells, which remains to be further elucidated.

In conclusion, GSDME mediates pyroptosis downstream of the ROS/JNK/Bax-mitochondrial apoptotic pathway and caspase-3/-9 activation. Our study indicated that GSDME-dependent pyroptosis is an unrecognized mechanism by which lobaplatin eradicates neoplastic cells, which may have important implications for the clinical and optical application of anticancer therapeutics.

## Supplementary information

Supplemental Figure Legend

Figure S1

Figure S2

Figure S3

Figure S4

Figure S5

Figure S6
