# Supplementary material for: Cleavage of GSDME by caspase-3 determines lobaplatin-induced pyroptosis in colon cancer cells
Source: Cell Death Dis. 2019 Feb 25;10(3):193. doi: 10.1038/s41419-019-1441-4 (PMC6389936; doi:10.1038/s41419-019-1441-4)
Supplement: Supplementary file 1 — Supplemental Figure Legend [file 41419_2019_1441_MOESM1_ESM.docx]

Figure S1. The RIPK3 inhibitor GSK’872 did not reduce pyroptotic cell death. To distinguish pyroptosis from necroptosis, the RIPK3 inhibitor GSK’872 was used to block the necroptotic pathway. Representative bright-field microscopy images of HT-29 and HCT116 cells treated with lobaplatin in the presence or absence of GSK’872 (10 µM) (a). Gel images of GSDME-N expression levels in each group of HT-29 and HCT116 cells (b, c). All the data are presented as the mean ± SD from three independent experiments. *p < 0.05 vs. control using one-way ANOVA.

Figure S2. GSDME-deficient Caco-2 cells did not show pyroptotic features upon exposure to lobaplatin. Gel images of GSDMD (a) and GSDME (b) expression levels in HT-29, HCT116 and Caco-2 cells (a). Representative bright-field microscopy images of Caco-2 cells treated with lobaplatin (c). Scale bar, 50 μm. The percentage of annexin V and 7-AAD double-positive Caco-2 cells after lobaplatin treatment was detected by flow cytometry (d). The release of IL-1β and LDH from Caco-2 cells treated with lobaplatin was measured by ELISA (e). Gel images of GSDME-N and cleaved CASP-3/-9 expression levels in Caco-2 cells overexpressing Bax (f). Representative bright-field microscopy images of Caco-2 cells overexpressing Bax (g). Scale bar, 50 μm. The release of IL-1β and LDH from Caco-2 cells overexpressing BAX was measured by ELISA (h). All the data are presented as the mean ± SD from three independent experiments. *p < 0.05 vs. control using Student’s t test.

Figure S3. GSDMD, rather than GSDME, is cleaved in LPS-induced pyroptosis in colon cancer cells. Representative bright-field microscopy images of HT-29 (a) and HCT116 (b) cells treated with LPS. Red arrowheads indicate large bubbles emerging from the plasma membrane. Scale bar, 50 μm. The release of IL-1β and LDH from HT-29 (c) and HCT116 (d) cells treated with LPS was measured by ELISA. Gel images of GSDMD-C, GSDME-N and cleaved CASP-1 expression levels in HT-29 (e) and HCT116 (f) cells treated with LPS. All the data are presented as the mean ± SD from three independent experiments. *p < 0.05 vs. control using Student’s t test.

Figure S4. GSDME knockout did not affect the apoptotic character of cell death induced by lobaplatin. The percentage of tunnel-positive cells in HT-29 (a) and HCT116 (b) cells or xenograft tumours formed by HT-29 cells (c) treated with lobaplatin in the presence or absence of GSDME knockout were detected by tunnel analysis.

Figure S5. Caspase-9 was responsible for GSDME and caspase-3 cleavage induced by Bax overexpression. Gel images of GSDME-N and cleaved CASP-3 expression levels in HT-29 (a) and HCT116 (b) cells overexpressing Bax in the presence or absence of caspase-9 knockdown. The release of IL-1β and LDH from HT-29 (c) and HCT116 (d) cells was measured by ELISA. All the data are presented as the mean ± SD from three independent experiments. *p < 0.05 vs. control using one-way ANOVA.

Figure S6. Transfection efficiency for siRNA, lentiviral shRNA-GSDMD vectors or Bax-overexpressing plasmid was identified by western blotting. Gel images of NLRP3, GSDMD, CASP-1/-3/-6/-7/-9 and BAX in HT-29 and HCT116 cells transfected with siRNA targeting NLRP3 (a) or CASP-1/-3/-6/-7/-9 (c, e), lentiviral shRNA-GSDMD vectors (b) and Bax-overexpressing plasmids(d).
