# Supplementary figures and images for: Cleavage of GSDME by caspase-3 determines lobaplatin-induced pyroptosis in colon cancer cells
Source: Cell Death Dis. 2019 Feb 25;10(3):193. doi: 10.1038/s41419-019-1441-4 (PMC6389936; doi:10.1038/s41419-019-1441-4)

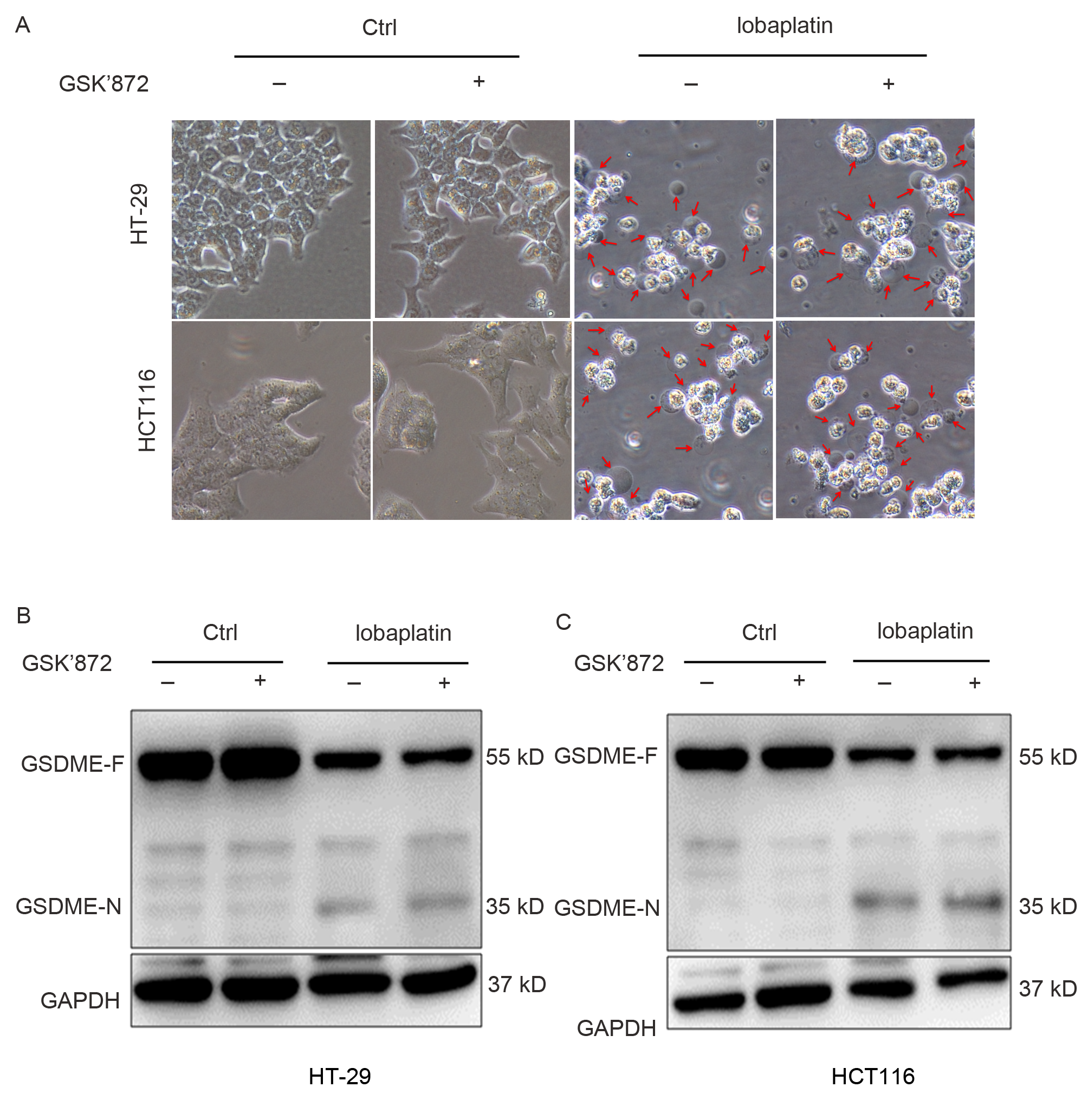

Supplement: Supplementary file 2 — Figure S1 [file 41419_2019_1441_MOESM2_ESM.tif]

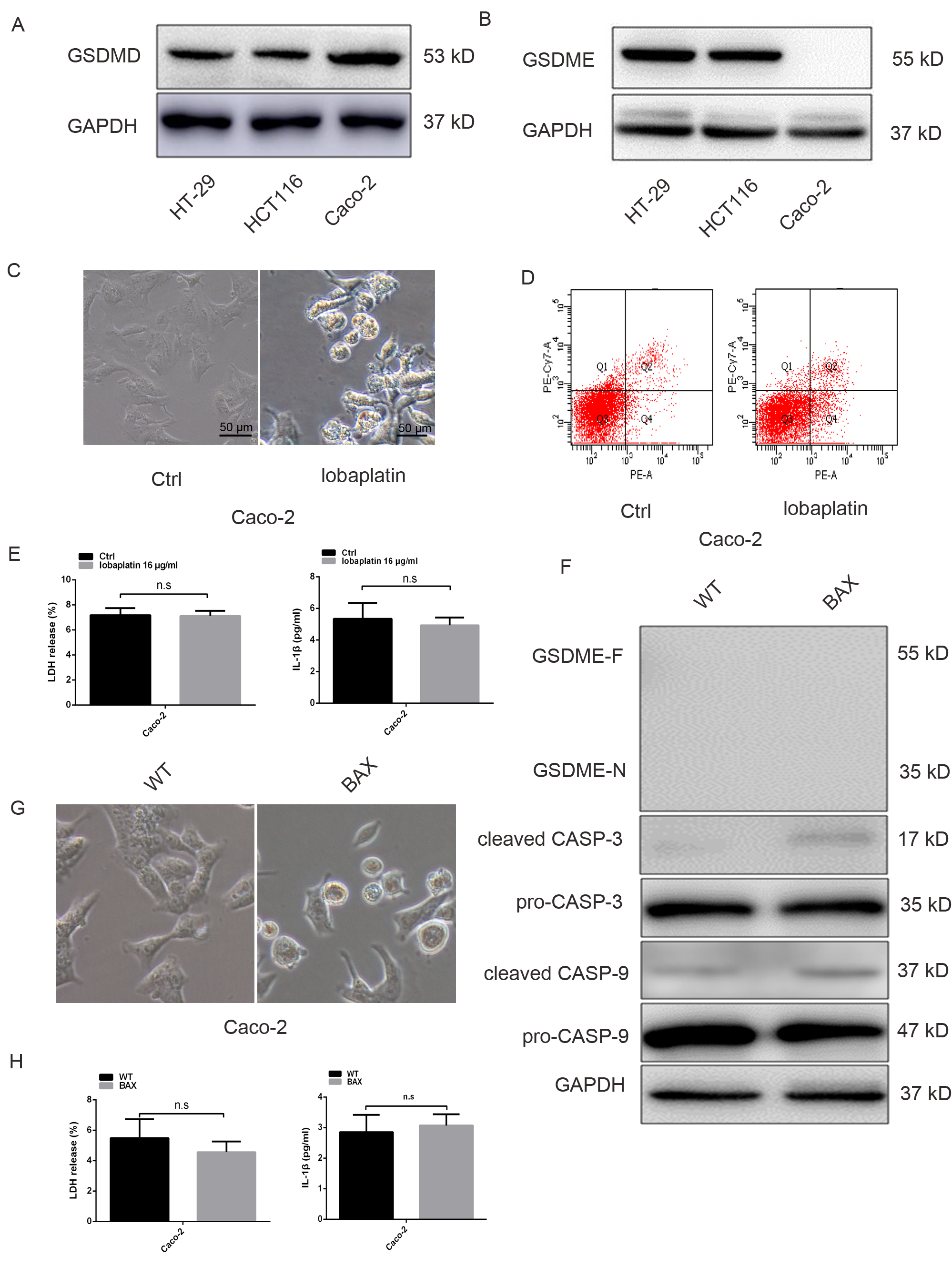

Supplement: Supplementary file 3 — Figure S2 [file 41419_2019_1441_MOESM3_ESM.tif]

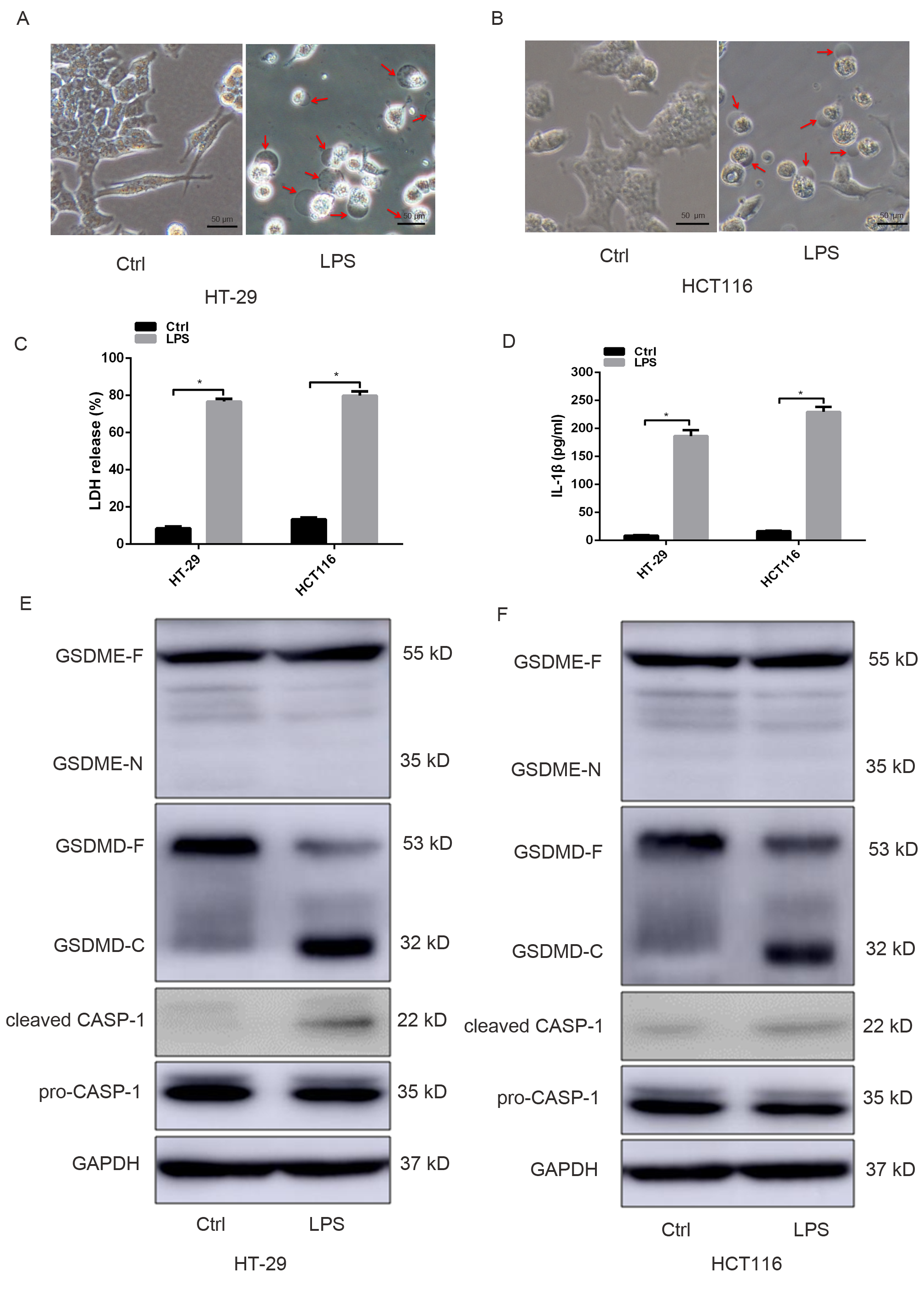

Supplement: Supplementary file 4 — Figure S3 [file 41419_2019_1441_MOESM4_ESM.tif]

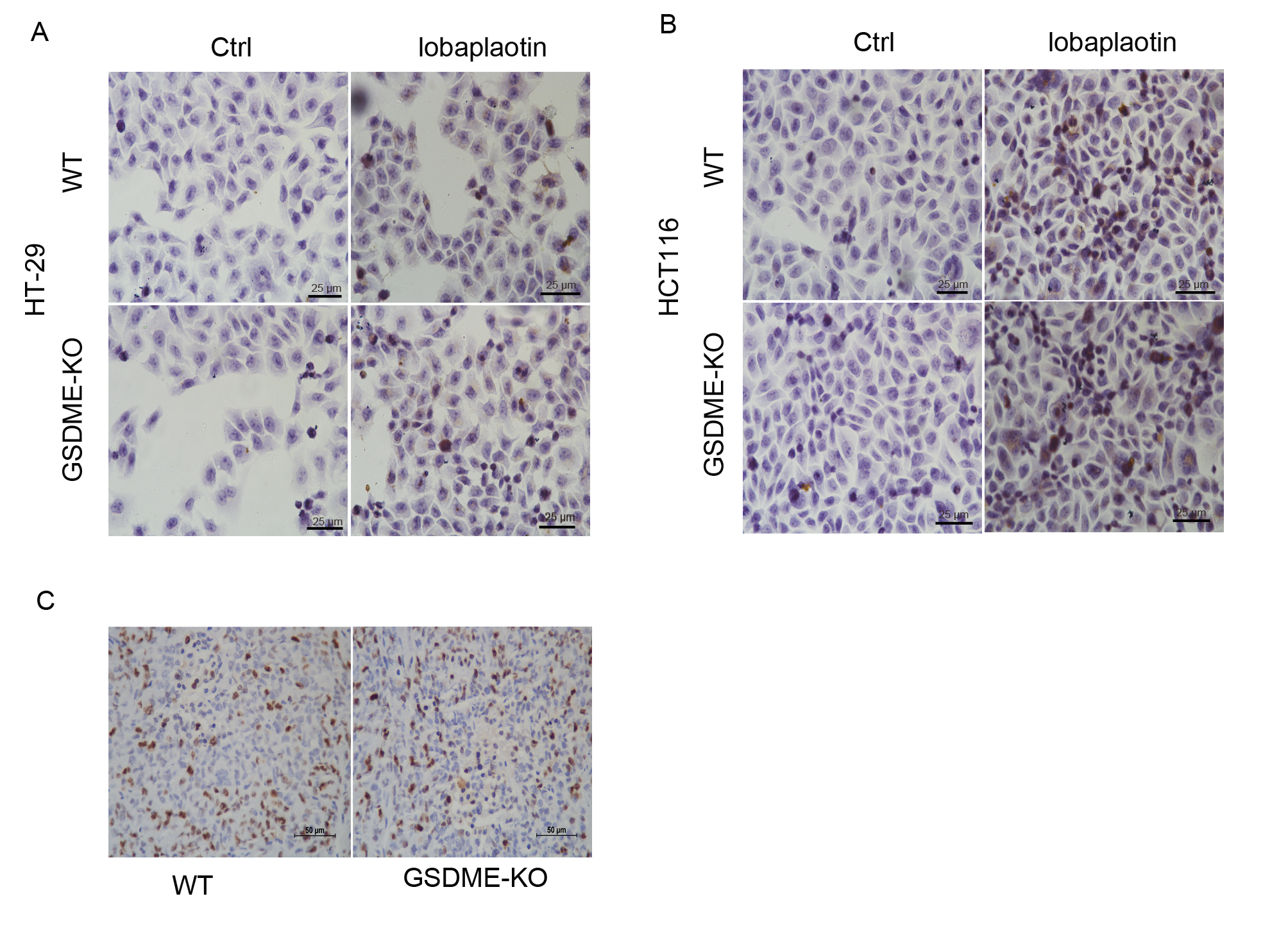

Supplement: Supplementary file 5 — Figure S4 [file 41419_2019_1441_MOESM5_ESM.tif]

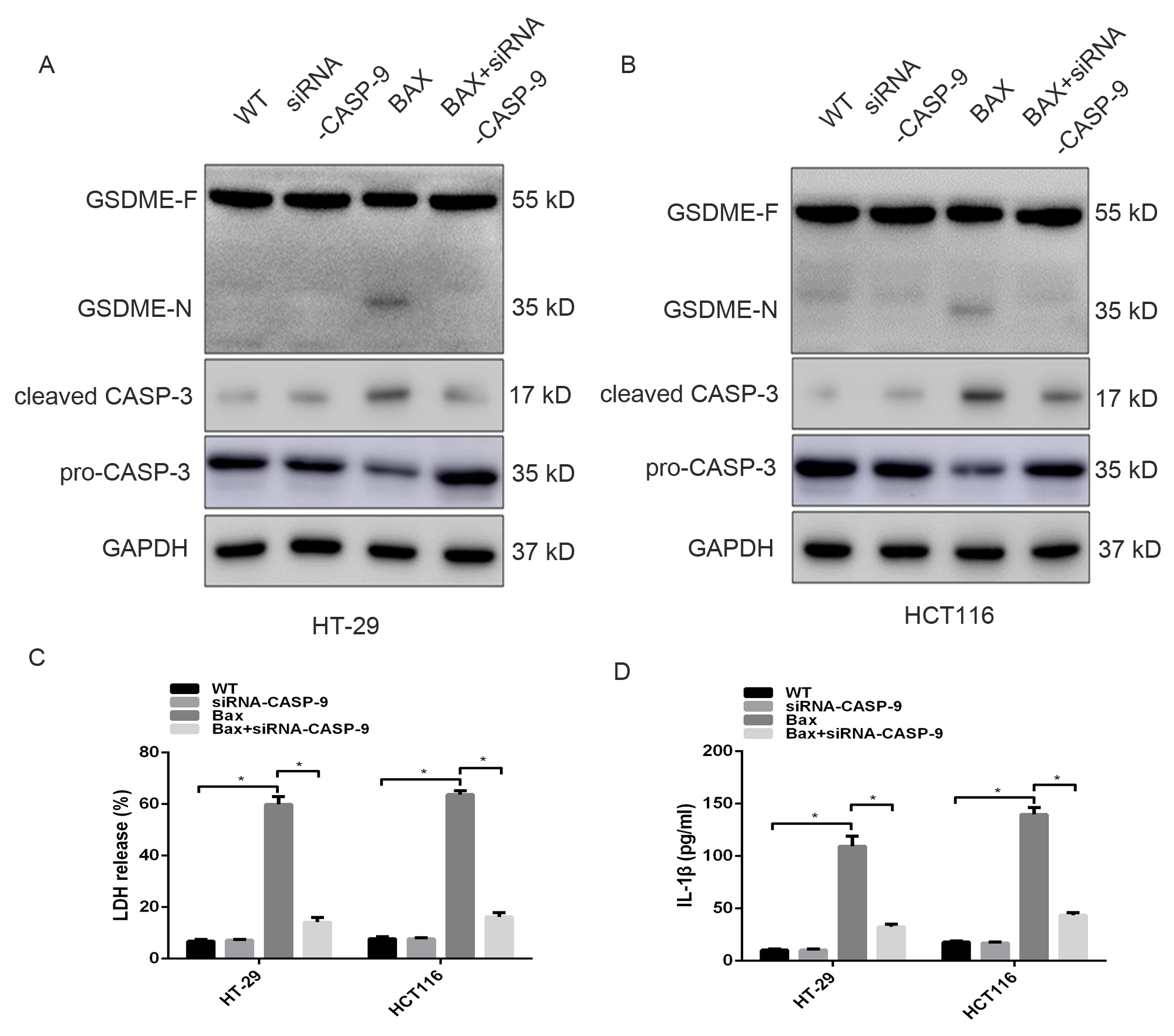

Supplement: Supplementary file 6 — Figure S5 [file 41419_2019_1441_MOESM6_ESM.tif]

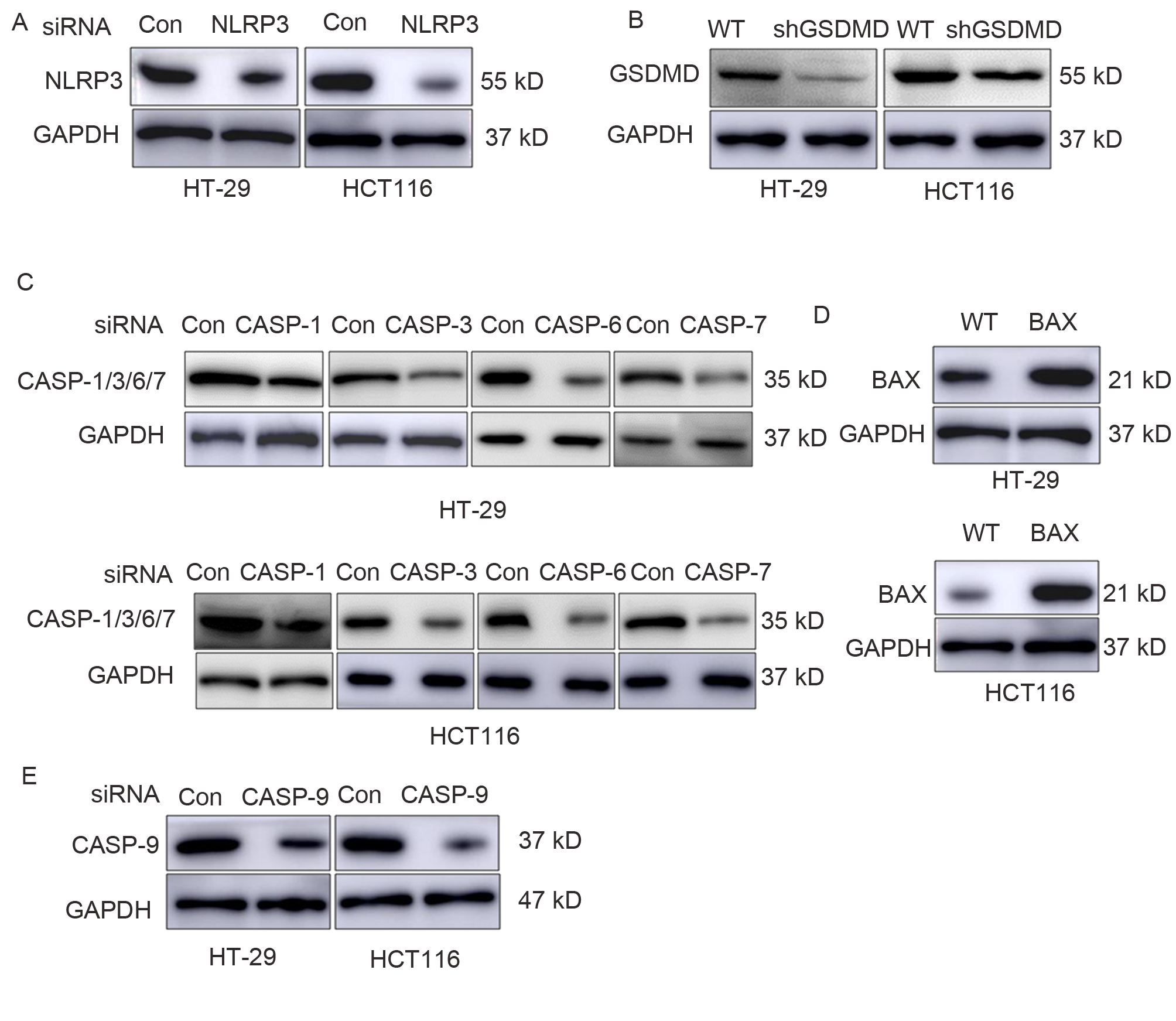

Supplement: Supplementary file 7 — Figure S6 [file 41419_2019_1441_MOESM7_ESM.tif]
